# *SHLD2* loss is a synthetic vulnerability to Polθ inhibition combined with radiotherapy

**DOI:** 10.1126/sciadv.aeb4508

**Published:** 2026-06-12

**Authors:** Gonzalo Rodriguez-Berriguete, Purusotha Thambiayah, Alessandro Cicconi, Nicole Machado, Celia Gotorbe, David Nderitu, Wei-Chen Cheng, Gerissa Fowler, Marie Laure Boursier, Aurora Cerutti, Vera Grinkevich, Bethany Rebekah Hill, Katjuša Koler, Sophie Alice Langdon, Jayesh B. Majithiya, Suraj Menon, Shaun Moore, Joana Neves, Natalie M. Palmer-Deverill, Eeson Rajendra, Marina Roy-Luzarraga, Asmita Thapa, Robert A. Heald, Graeme C. M. Smith, Helen M. R. Robinson, Marco Ranzani, Geoff S. Higgins

**Affiliations:** ^1^ Department of Oncology, University of Oxford, Oxford, UK.; ^2^ Artios Pharma, Babraham Research Campus, Cambridge, UK.

## Abstract

Inhibition of DNA polymerase theta (Polθ), an essential enzyme for repairing DNA double-strand breaks (DSBs) via microhomology-mediated end joining (MMEJ), has proven to be an exquisitely effective monotherapy in HR-deficient tumor models. In addition, Polθ inhibition (Polθi) can induce tumor-selective radiosensitization, but unlike its monotherapy use, no clinically actionable biomarkers have yet been identified to predict this effect. Here, we profiled 54 cancer cell lines and found that Polθi induces substantial radiosensitization in most models, although with marked variability not explained by indicators of Polθ activity. To pinpoint molecular determinants of radiosensitization by Polθi, we performed a CRISPR knockout screen which revealed loss of the TP53BP1/Shieldin pathway component *SHLD2* (*FAM35A*) as a vulnerability to Polθi combined with RT. We found that *SHLD2* is deleted in a subset of human prostate cancers, frequently alongside *PTEN* loss, an adverse prognostic factor. We demonstrated that *SHLD2* loss not only increases sensitivity to RT alone, as reported previously, but also enhances the radiosensitizing effect of Polθi, independently of *PTEN* status and without requiring HR deficiency. Moreover, our findings support a model in which *SHLD2* deficiency increases Polθ dependence following RT, with Polθ activity limiting DSB accumulation and chromosomal instability, via a compensatory mechanism independent of canonical MRE11/CtIP-mediated DNA end resection. In summary, we found that *SHLD2* loss is a collateral vulnerability that can be exploited through combined treatment with Polθi and RT.

## Teaser

Loss of *SHLD2* creates a clinically actionable vulnerability to combined Polθ inhibition (Polθi) and radiotherapy (RT).

## INTRODUCTION

Radiotherapy (RT) plays a key role in the treatment of many solid tumors. When given with curative intent, RT is often combined with conventional chemotherapies to sensitize tumors to ionizing radiation. However, these effects are not tumor specific, and the benefit of these treatments in improving tumor control is offset by side effects caused by increased damage to surrounding normal tissues (*1*). Therefore, there is major interest in combining RT with drugs that exert a radiosensitizing effect selectively in tumor cells. In this regard, we have previously reported that pharmacological inhibition of Polθ is an effective and safe approach for tumor-selective radiosensitization in preclinical models (*2*).

Polθ (encoded by the *POLQ* gene) is a type A DNA polymerase that also contains a helicase domain and plays a critical role in the repair of DNA double-strand breaks (DSBs) by microhomology-mediated end joining (MMEJ), an error prone repair pathway (*3*). According to current models, during MMEJ repair, MRE11/CtIP-mediated initiation of 5′ → 3′ resection at the DSB generates 3′ overhangs, exposing short, complementary microhomologies (*4*). The helicase domain of Polθ then facilitates the annealing of these microhomologies, and its polymerase domain fills in and seals the resulting gaps (*5*, *6*). This error-prone repair leaves characteristic genomic “scars” at the DSB site, consisting of short deletions flanked by microhomologies, which reflect the mutagenic signature of Polθ (*7*).

Polθ is synthetically lethal with components of the homologous recombination (HR) pathway, such as *BRCA1* and *BRCA2* (*8*–*10*). In addition, Polθi has been shown to enhance the antitumor effect of poly(ADP-ribose) polymerase inhibition (PARPi) in HR-deficient cancer cells (*8*, *10*–*12*). This has led to ongoing clinical testing of Polθi in patients with HR-deficient cancers as a monotherapy and in combination with PARPi (NCT05898399, NCT04991480, NCT06077877, NCT05687110, NCT06666270, NCT06545942, and NCT06560632). In contrast, we have previously demonstrated that HR deficiency is not required for Polθi-induced radiosensitization (*2*). Therefore, the combination of Polθi with RT may benefit a broader patient population than Polθi alone or in combination with PARPi. Nonetheless, it remains unclear how variability among tumors influences the radiosensitizing effect of Polθi, and to date, no clinically actionable molecular markers exist to identify which patients could benefit the most from Polθi combined with RT. Addressing these gaps will be crucial for the effective clinical translation of this combination treatment.

## RESULTS

### Polθi-induced radiosensitization varies across cancer cell lines and does not correlate with indicators of Polθ activity

**Fig. 1.**
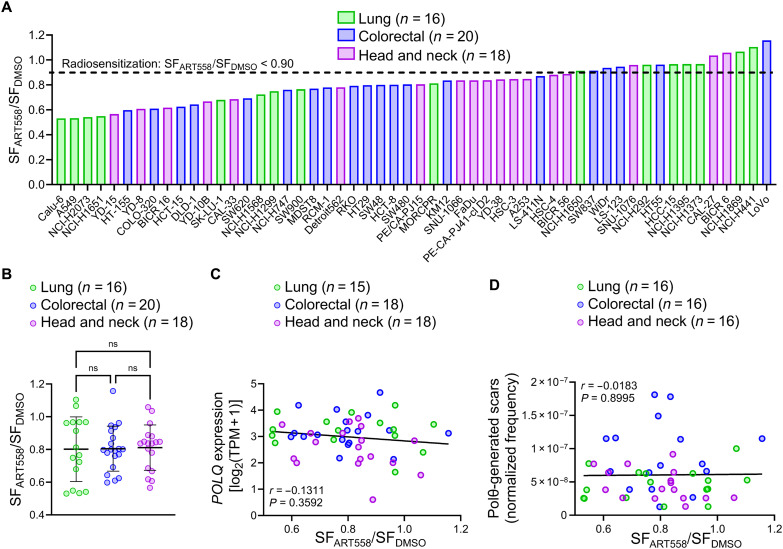
Polθi-induced radiosensitization varies across cancer cell lines and does not correlate with indicators of Polθ activity. (**A**) Radiosensitization by Polθi in a panel of lung, colorectal, and head and neck cancer cell lines, estimated as the surviving fraction (SF) ratios of ART558- to DMSO-treated cells (SF_ART558_/SF_DMSO_). (**B**) SF_ART558_/SF_DMSO_ values stratified by tumor type (mean ± SD). (**C**) Correlation between *POLQ* expression and the magnitude of radiosensitization by Polθi. TPM, transcripts per million. (**D**) Correlation between the frequency of Polθ-generated scars per exomic base pair and the magnitude of radiosensitization by Polθi. Dots represent individual cell lines. Statistical significance in (B) was calculated using one-way analysis of variance (ANOVA). Correlation coefficients (*r*) in (C) and (D) were calculated using the Spearman’s method. The black lines in (C) and (D) are regression lines. ns, not significant.

Our previous work showed that small-molecule, allosteric inhibitors of Polθ’s polymerase domain—ART558 and ART899—are effective, tumor-selective radiosensitizers in preclinical models (*2*). To interrogate to what extent tumor cell heterogeneity affects radiosensitization by Polθi and to explore its potential for broad clinical application, we evaluated ART558-mediated radiosensitization in a panel of 54 lung, colorectal, and head and neck cancer cell lines. As shown in Fig. 1A, the magnitude of radiosensitization by Polθi varied considerably (SF_ART558_/SF_DMSO_ range: 0.53 to 1.16), with 72% of the cancer cell lines showing substantial radiosensitization (SF_ART558_/SF_DMSO_ < 0.9). The average radiosensitization by Polθi did not differ among the cell lines representative of three tumor types (Fig. 1B). Moreover, the radiosensitizing effect of Polθi did not correlate with *POLQ* expression or the frequency of Polθ-generated DNA scars (*7*)—putative indicators of Polθ activity (Fig. 1, C and D, and fig. S1, A and B). These results highlight the broad potential of Polθi as a radiosensitization strategy. Nonetheless, the pronounced variability in radiosensitization, along with the lack or limited response observed in 28% of the models, underscores the need to identify determinants of Polθi-induced radiosensitization to maximize the clinical benefit of combining Polθi with RT.

### A CRISPR knockout screen identifies genetic modulators of Polθi-induced radiosensitization

**Fig. 2.**
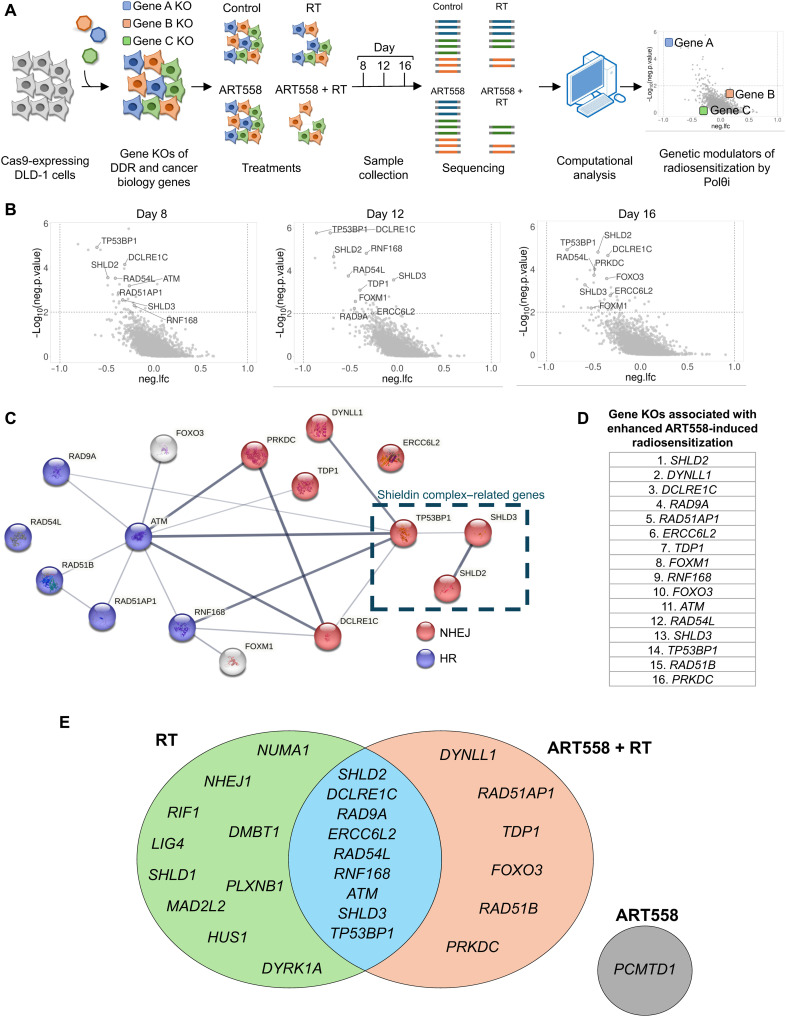
A CRISPR KO screen identifies genetic modulators of the radiosensitizing effect of Polθi. (**A**) Outline of the CRISPR KO screen to identify genetic modulators of Polθi-induced radiosensitization. The timeline is described in fig. S2A. DDR, DNA damage response. (**B**) Volcano plots highlighting the specific genes whose KO synergized with combined Polθi (ART558) and RT (4 × 0.5 Gy) in at least two of the time points and/or RT schedules. For the volcano plots corresponding to the 4 × 1 Gy schedule, see fig. S2F. neg.lfc, negative log fold change. (**C**) Network of protein-protein interactions among the genes whose KO was associated with enhanced Polθi-induced radiosensitization, generated with the STRING database. The thickness of the connecting lines indicates the strength of the data supporting the interaction. Genes involved in HR and NHEJ have been highlighted in blue and red, respectively. (**D**) Rank of gene KOs associated with sensitization to ART558 + RT identified in the CRISPR screen. (**E**) Venn diagram showing the top-ranking gene KOs associated with sensitization to the monotherapies (RT and ART558) and the combination treatment (ART558 + RT). Ranked gene lists with further details are provided in data S1 (D and E).

To identify genetic determinants of the response to Polθi and RT, we conducted a CRISPR knockout (KO) screen in Cas9-expressing DLD-1 cells targeting 2776 genes involved in the DNA damage response and cancer biology (Fig. 2A, fig. S2A, and data S1A). Screen quality controls confirmed optimal transduction and Cas9 cutting efficiency, adequate representation, and high specificity and sensitivity in the detection of essential genes (fig. S2, B to E). Using Model-based Analysis of Genome-wide CRISPR/Cas9 Knockout (MAGeCK) analysis of relative single guide RNA (sgRNA) abundance at the different time points posttreatment, we identified several specific gene KOs conferring significant sensitization to RT, Polθi, and the combination treatment (Fig. 2, B to E; fig. S2F; and data S1, D and E).

Among the specific gene KOs conferring enhanced sensitization to the combination of Polθi and RT—exceeding the expected additive effects of either agent alone (see Materials and Methods)—we identified factors primarily involved in DSB repair via nonhomologous end joining (NHEJ) and HR (Fig. 2, B to E; fig. S2, F and G; and data S1E). This is consistent with the previously reported role of Polθ in driving DSB repair when these pathways are compromised (*8*, *9*, *13*, *14*). Three physically interacting proteins of the TP53BP1/Shieldin pathway were identified as suppressors of the radiosensitizing effect of Polθi: *SHLD2* (*FAM35A*), *SHLD3* (*RINN1*), and *TP53BP1* (Fig. 2, B to E; fig. S2, F and G; and data S1E). Of these, *SHLD2* was the top-ranking gene, with its loss consistently showing synergistic negative enrichment in the RT and Polθi treatment arm at the two radiation doses across all experimental time points (Fig. 2, D and E, and data S1E).

### *SHLD2* is frequently lost in patients with prostate cancer

**Fig. 3.**
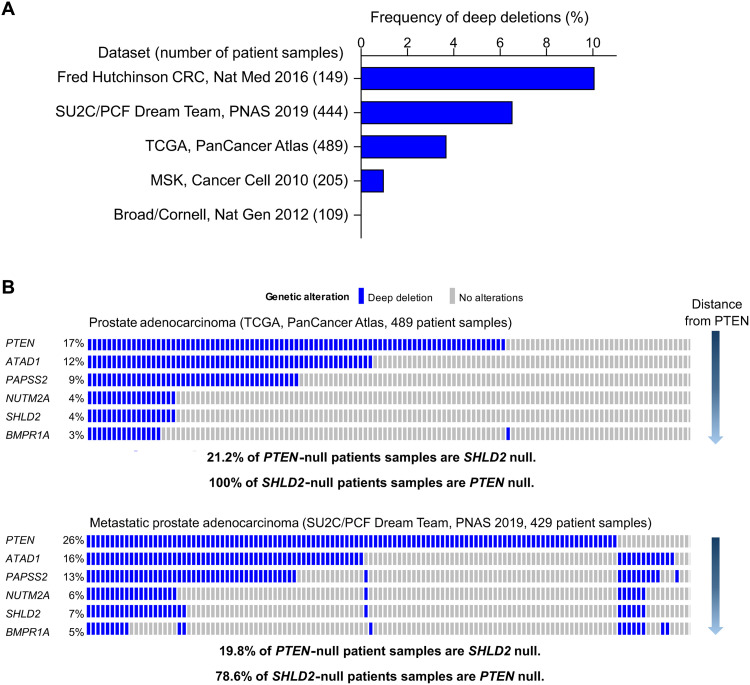
*SHLD2* is frequently lost in human prostate tumors. (**A**) Proportion of patient samples with homozygous *SHLD2* deletions in the indicated prostate cancer datasets. Numbers in brackets indicate the total number of patient samples with CNV data available. (**B**) Proportions of homozygous deletions in *PTEN* and neighbor genes. Each bar within the same column represents the same patient sample, enabling visualization of co-occurring deletions.

We next interrogated databases of large prostate cancer cohorts (*15*–*20*) and found that *SHLD2* is frequently lost in prostate cancer, with up to 10% of patient samples displaying homozygous deletions in this gene (Fig. 3A). Notably, ~4% of patients in The Cancer Genome Atlas (TCGA) cohort (*19*, *20*)—which comprises primary, localized prostate tumors which are typically treated with curative-intent RT—also exhibited homozygous *SHLD2* deletions (Fig. 3A).

*SHLD2* is located less than 1 mega–base pair (Mbp) away from *PTEN* (fig. S3), a tumor suppressor gene that is frequently deleted in prostate cancer and whose loss is associated with adverse outcomes (*21*). We found that homozygous *SHLD2* deletions occur in ~20% of tumors with homozygous *PTEN* deletions and that between 79 and 100% of tumors with homozygous *SHLD2* deletions harbor homozygous *PTEN* deletions (Fig. 3B). In addition, we observed that codeletion rates among genes flanking *PTEN* decrease with increasing distance from *PTEN* (Fig. 3B and fig. S3). Together, these findings indicate that *SHLD2* loss may constitute a collateral therapeutic vulnerability in prostate cancer that can be exploited through the combination of Polθi and RT.

In view of its potential clinical relevance, and as it emerged in the screen as the top gene associated with enhanced Polθi-induced radiosensitization, we prioritized *SHLD2* for further investigation. Since *SHLD2* depletion was also associated with enhanced sensitivity to RT alone, combining RT with Polθi would be expected to produce a particularly strong absolute treatment effect in *SHLD2*-deficient tumors, due to both high baseline sensitivity to RT and the synergistic effect of Polθi when combined with RT. This further supported prioritizing *SHLD2* for further investigation.

### *SHLD2* loss synergizes with Polθi combined with RT independent of *PTEN* status in vitro

Loss of TP53BP1/Shieldin has previously been shown to confer PARPi resistance and to synergize with Polθi monotherapy specifically in HR-deficient cells (*10*, *12*). It is important to note that the DLD-1 cells used in our CRISPR KO screen are HR proficient (*10*). Therefore, our observation that TP53BP1/Shieldin deficiency may sensitize HR-proficient cells to combined Polθi and RT represents a novel finding.

**Fig. 4.**
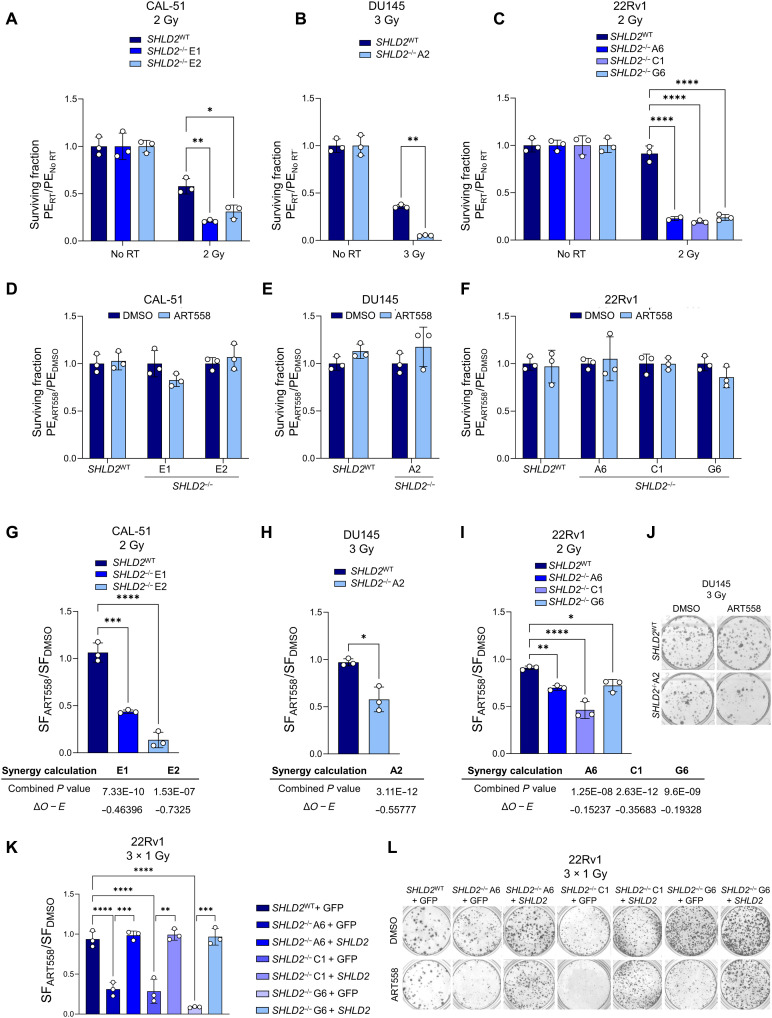
*SHLD2* loss synergizes with combined Polθi and RT in vitro. Colony-forming capacity of *SHLD2*^WT^ versus *SHLD2*^−/−^ CAL-51, DU145, and 22Rv1 cells treated with: (**A** to **C**) RT alone, normalized to unirradiated controls; (**D** to **F**) 4 μM Polθi ART558 alone for 14 days, normalized to dimethyl sulfoxide (DMSO)–treated control; (**G** to **I**) 4 μM Polθi ART558 and RT, expressed as the SF ratios of ART558- to DMSO-treated cells, to show the radiosensitizing effect of Polθi. Synergy calculations for each *SHLD2*^−/−^ clone was performed according to Materials and Methods. (**J**) Representative colony formation assays of *SHLD2*^WT^ and *SHLD2*^−/−^ DU145 cells treated with RT and either DMSO or ART558. (**K**) Colony formation capacity of the *SHLD2*^WT^ versus *SHLD2*^−/−^ 22Rv1 models transduced with either a vector expressing *SHLD2* or a GFP control vector. Radiosensitization by Polθi is shown as the SF ratios of ART558- to DMSO-treated cells. (**L**) Representative colony formation assays from (K). Mean ± SD of three technical replicates (representative of two independent experiments). Statistical tests: (A to C) two-way ANOVA and Tukey’s post hoc test: *P < 0.05, **P < 0.01, and ****P < 0.0001; (D to F) multiple unpaired *t* tests; (G to I and K) one-way ANOVA and Dunnett’s post hoc test for CAL-51 and 22Rv1 graphs and Welch’s *t* test for DU145 graphs: *P < 0.05, **P < 0.01, ***P < 0.001, and ****P < 0.0001.

Given the clinical relevance of *SHLD2* loss in prostate cancer, we generated *SHLD2* KO clones (*SHLD2^−/−^*) from the HR-proficient (*22*) and *PTEN*-expressing (*23*) prostate cancer cell lines DU145 (clone A2) and 22Rv1 (clones A6, C1, and G6) (fig. S4A), to validate *SHLD2* loss as both a therapeutic vulnerability and a potential predictive biomarker of Polθi-mediated radiosensitization. Since, according to our codeletion analysis (Fig. 3B), most prostate cancer tumors with homozygous *SHLD2* deletions are also *PTEN* null, we knocked out *SHLD2* in the *PTEN*-null (*24*), HR-proficient (*22*) breast cancer cell line CAL-51 (clones E1 and E2) (fig. S4A), to rule out an effect of *PTEN* loss on Polθi-induced radiosensitization. Compared to their parental counterparts, the *SHLD2^−/−^* clones were more sensitive to RT alone in vitro (Fig. 4, A to C), in line with the role of the TP53BP1/Shieldin pathway in promoting DSB repair by NHEJ (*25*–*27*) and with the results of our screen (data S1D). Continuous treatment with ART558 alone for 14 days did not affect the colony-forming ability of the *SHLD2^−/−^* cells (Fig. 4, D to F), confirming that these HR-proficient models are not susceptible to Polθi monotherapy, unlike what was reported previously for HR-deficient, TP53BP1/Shieldin-deficient cells (*10*, *12*). However, compared to the *SHLD2^WT^* parental lines, the extent of Polθi-induced radiosensitization with ART558 was significantly higher in the *SHLD2^−/−^* clones, irrespective of the *PTEN* status (Fig. 4, G to J).

Since radiosensitivity varies depending on the cell cycle phase in which cells are irradiated, we analyzed the cell cycle distribution of both DU145 and 22Rv1 cells collected at the time of radiation. No differences in the proportions of cells across cell cycle phases were observed between the *SHLD2^WT^* and *SHLD2^−/−^* cells, nor between vehicle- and ART558-treated conditions, indicating that the observed radiosensitization is not attributable to differences in cell cycle distribution (fig. S4, B and C). Reconstitution of *SHLD2* expression in the 22Rv1 *SHLD2^−/−^* clones reversed the radiosensitizing effect of Polθi, demonstrating the direct role of *SHLD2* in modulating this response (Fig. 4, K to L, and fig. S4D). Furthermore, depletion of *POLQ* in *SHLD2*^−/−^ A2 cells both sensitized these cells to RT alone and completely abrogated the radiosensitizing activity of ART558, confirming that this inhibitor specifically targets Polθ (fig. S4, E and F), as we previously reported (*2*, *10*).

To determine whether this effect extended to other components of the TP53BP1/Shieldin axis, we transiently targeted *TP53BP1*, *RIF1*, *MAD2L2*, *SHLD1*, and *SHLD3* using small interfering RNAs (siRNAs) and assessed sensitivity to combined RT and Polθi. Targeting of *TP53BP1* and *MAD2L2* enhanced Polθi-mediated radiosensitization, phenocopying *SHLD2* loss. In contrast, although targeting of *RIF1*, *SHLD1*, or *SHLD3* increased sensitivity to RT alone, it did not further augment the radiosensitizing effect of Polθi (fig. S5, A and B). These findings indicate different functional contributions of individual TP53BP1/Shieldin components in modulating the response to combined RT and Polθi.

### *SHLD2*-deficient cells display compensatory dependence on Polθ to limit DSB accumulation and genomic instability following RT

**Fig. 5.**
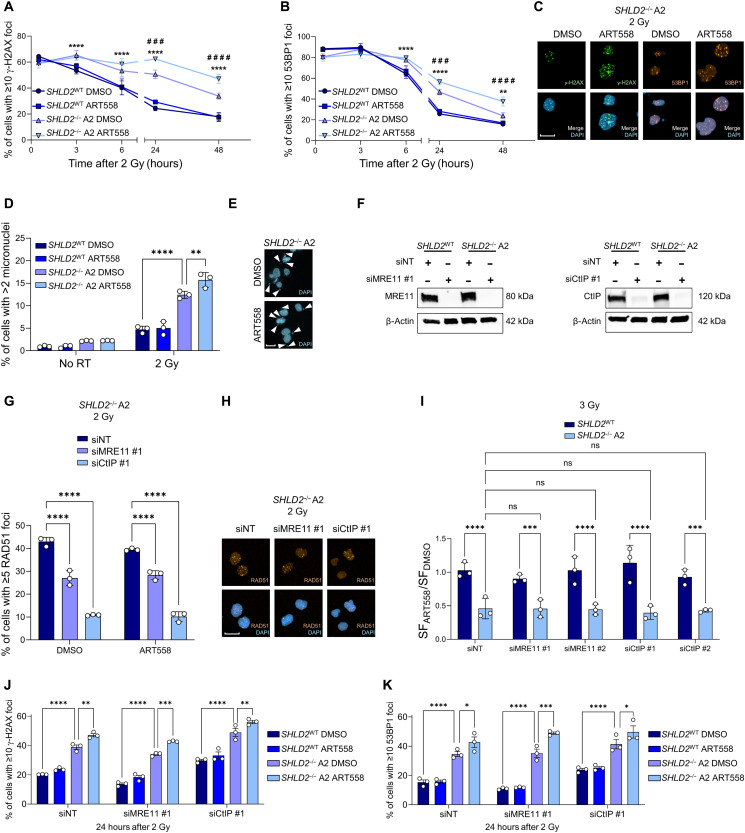
*SHLD2*^−/−^ cells rely more on Polθ to prevent RT-induced DSB accumulation and chromosomal instability, independently of MRE11/CtIP-mediated resection. *SHLD2*^WT^ and *SHLD2*^−/−^ A2 DU145 cells were treated with 4 μM Polθi ART558 and the indicated RT doses. (**A**) γH2AX and (**B**) 53BP1 foci dynamics, assessed by fluorescence microscopy. (**C**) Images from (A) and (B) at 24 hours post-RT. (**D**) Micronuclei formation 48 hours after RT, assessed by fluorescence microscopy. (**E**) Images of RT-treated *SHLD2*^−/−^ A2 cells from (D). Arrowheads indicate micronuclei. (**F**) Western blots demonstrating depletion of MRE11 and CtIP at the time of RT, after transfection with the siMRE11 #1 and siCtIP #1 siRNAs. A nontargeting siRNA was used as a control (siNT). See fig. S6G for siMRE11 #2 and siCtIP #2 Western blots. (**G**) RAD51 foci at 3 hours post-RT upon siRNA-mediated depletion of MRE11 and CtIP, assessed by fluorescence microscopy. (**H**) Images of RT-treated *SHLD2*^−/−^ A2 cells from (G). (**I**) Extent of radiosensitization by Polθi upon siRNA-mediated depletion of MRE11 and CtIP using two different siRNA strands (#1 and #2) assessed by colony formation assay. (**J**) γH2AX and (**K**) 53BP1 foci assessed 24 hours post-RT following transfection with the siMRE11 #1 and siCtIP #1. Data are representative of three independent experiments, except (G) and (H) which are representative of two independent experiments, showing mean ± SD from triplicate wells. Statistical significance was calculated with two-way ANOVA and Tukey’s post hoc test. In (A) and (B), hashes and asterisks refer to the comparison of DMSO-treated *SHLD2*^−/−^ versus ART558-treated *SHLD2*^−/−^ cells and DMSO-treated *SHLD2*^WT^ versus DMSO-treated *SHLD2*^−/−^ cells, respectively. # or *P < 0.05, ## or **P < 0.01, ### or ***P < 0.001, and #### or ****P < 0.0001. Scale bars, 20 μm (C, E, and H). DAPI, 4′,6-diamidino-2-phenylindole.

To investigate the mechanism by which *SHLD2* loss confers sensitivity to Polθi and RT, we assessed the dynamics of DSB markers following RT in DU145 *SHLD2^WT^* and *SHLD2^−/−^* A2 cells, using fluorescence microscopy. Compared to the *SHLD2^WT^* cells, the *SHLD2^−/−^* cells exhibited increased levels of γH2AX, 53BP1, pATM, and RAD51 nuclear foci, becoming significant as early as 3 to 6 hours post-RT (Fig. 5, A to C, and fig. S6, A to F). A further increase in these DSB markers was consistently observed upon Polθi treatment at 24 to 48 hours after RT in the *SHLD2^−/−^* A2 DU145 cells, but not in their parental *SHLD2^WT^* counterparts (Fig. 5, A to C, and fig. S6, A to F). We also determined the induction of cytoplasmic micronuclei in the *SHLD2^WT^* and *SHLD2^−/−^* A2 DU145 cell lines after RT, as a marker of genomic instability (*28*). In line with the DSB foci data, the proportion of RT-induced micronuclei was higher in the *SHLD2^−/−^* cells than in the parental cells (Fig. 5, D and E). Moreover, further induction of micronuclei after RT was observed only in the *SHLD2^−/−^* cells treated with Polθi (Fig. 5, D and E). These results demonstrate that *SHLD2*-deficient cells are more reliant on Polθ to prevent RT-induced DSB accumulation and chromosomal instability.

### Radiosensitization by Polθi in *SHLD2*-deficient cells does not depend on MRE11/CtIP-mediated DNA end resection

The TP53BP1/Shieldin pathway has been implicated in counteracting DNA end resection at DSBs, thereby directing DNA repair toward NHEJ and suppressing HR (*4*, *27*, *29*–*34*). Since the single-stranded DNA (ssDNA) overhangs generated by MRE11/CtIP during resection are optimal substrates for Polθ-driven MMEJ (*4*, *35*–*38*), we hypothesized that *SHLD2* loss may increase the pool of resected DNA ends following RT, thereby providing additional substrates for Polθ-mediated MMEJ and enhancing the dependence on Polθ. Accordingly, we asked whether the radiosensitizing effect of Polθi in *SHLD2*-deficient cells depends on DNA end resection. To address this, we perturbed the short-range resection factors CtIP and MRE11 and assessed the radiosensitizing effect of Polθi in both DU145 and 22Rv1 *SHLD2^WT^* versus *SHLD2^−/−^* cells. Effective knockdown of MRE11 and CtIP (Fig. 5F and fig. S6, G and H) was accompanied by a reduction in RT-induced nuclear foci of the HR factor RAD51 (Fig. 5, G and H), indicating inhibition of DNA resection, which was particularly pronounced upon CtIP depletion. Knockdown of MRE11 and CtIP also increased sensitivity to RT alone in both DU145 *SHLD2*^WT^ and *SHLD2*^−/−^ cells (fig. S6I), demonstrating the functional impact of depleting these short-range resection factors on the RT response, in line with previous findings (*39*, *40*). However, we did not see a reversal of the radiosensitizing effect of Polθi upon depletion of MRE11 or CtIP in either the DU145 or the 22Rv1 *SHLD2^−/−^* clones (Fig. 5I and fig. S6J). In agreement, CtIP or MRE11 depletion did not reverse the accumulation of DSBs induced by Polθi after RT, as determined by γH2AX and 53BP1 foci assessment (Fig. 5, J and K). Together, these findings indicate that the enhanced sensitivity of *SHLD2*-deficient cells to Polθi following RT is not primarily dependent on MRE11/CtIP-mediated short-range resection.

### *SHLD2*-deficient tumors are highly sensitive to combined Polθi and radiation treatment in vivo

**Fig. 6.**
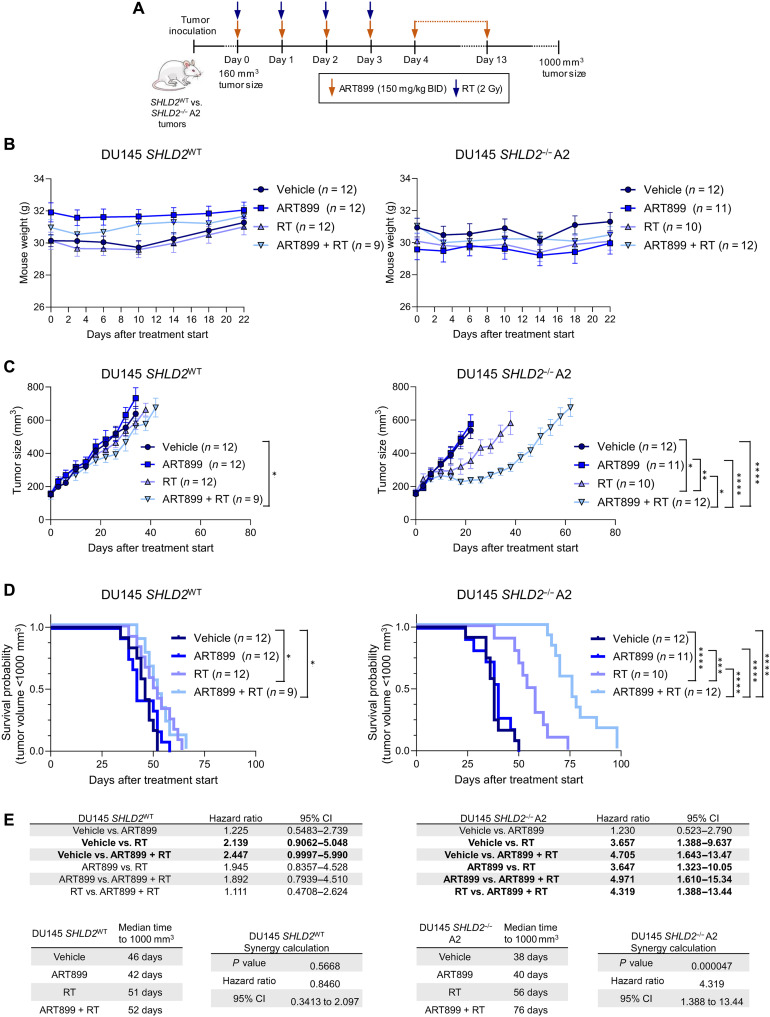
*SHLD2*-deficient tumors are highly sensitive to combined Polθi and radiation treatment in vivo. *SHLD2^WT^* and *SHLD2^−/−^* A2 DU145 xenografts in NRG mice treated with 4 × 2 Gy x-ray (RT) and/or the Polθi ART899 (150 mg/kg twice daily for 14 days). (**A**) Diagram of the experimental design. (**B**) Mouse weights. (**C**) Tumor growth kinetics of *SHLD2^WT^* and *SHLD2^−/−^ A2* DU145 xenografts. Mean ± SE. Unpaired Mann-Whitney *U* tests were used to assess differences in tumor size averages at day 22. *P < 0.05, **P < 0.005, and ****P < 0.0001. (**D**) Kaplan-Meier survival plot for a tumor threshold of 1000 mm^3^. Log-rank (Mantel-Cox) test: *P < 0.05, ***P < 0.0005, and ****P < 0.0001. (**E**) Tables show hazard ratios and 95% confidence intervals (CI) for the indicated comparisons, median times to reach a tumor size of 1000 mm^3^ across different treatments, and synergy analysis between RT and Polθi ART899 assessed according to details in Materials and Methods.

We next assessed the effect of Polθi combined with fractionated RT in NRG mice inoculated with the DU145 *SHLD2^WT^* and *SHLD2^−/−^* A2 cells (Fig. 6A). In this experiment, we used ART899, an analog of ART558 previously shown to display improved pharmaceutical properties, making it suitable for in vivo studies (*2*). Treatment with the Polθi ART899 in combination with fractionated RT was well tolerated, as indicated by the absence of significant changes in body weight (Fig. 6B). Consistent with our in vitro data, ART899 monotherapy did not affect the growth of either *SHLD2^WT^* or *SHLD2^−/−^* tumors, and the *SHLD2^−/−^* tumors were more sensitive to RT alone than their *SHLD2^WT^* counterparts (Fig. 6, C to E). Compared to RT alone, the addition of ART899 to RT induced a significant reduction in tumor growth and significant extension of survival in the *SHLD2^−/−^* xenografts, but not in *SHLD2^WT^* xenografts (Fig. 6, C to E). The synergy analysis demonstrated that, in *SHLD2^−/−^* xenografts, the observed survival with the combination treatment significantly exceeded the Bliss-predicted survival (hazard ratio > 1), confirming a synergistic interaction between ART899 and RT in SHLD2-deficient tumors in vivo. Overall, our results demonstrate that *SHLD2* loss renders tumors more susceptible to the combination of Polθi with RT in vivo.

## DISCUSSION

In the present study, using a comprehensive panel of cancer cell lines, we demonstrate that Polθi exerts a radiosensitizing effect in approximately three quarters of the models analyzed, underscoring the broad therapeutic potential of combining Polθi with RT. However, the extent of Polθi-induced radiosensitization varied considerably across the 54 cancer cell lines screened, with a nonnegligible subset of models exhibiting minimal or no response. This variability underscores the need for biomarkers to identify patients most likely to benefit from Polθi in combination with RT, which would significantly ease clinical translation and, ultimately, improve patient outcomes. The lack of correlation between indicators of Polθ activity and the extent of Polθi-induced radiosensitization prompted us to conduct a forward genetic CRISPR KO screen to uncover determinants of Polθi-induced radiosensitization.

In our CRISPR KO screen, components of the TP53BP1/Shieldin pathway (*SHLD2*, *SHLD3*, and *TP53BP1*) were among the genes whose loss was associated with enhanced radiosensitization by Polθi, with *SHLD2* as the highest-ranked hit. Here, we demonstrate that *SHLD2* loss markedly enhances tumor cell sensitivity to combined Polθi and RT in both cancer cell lines in vitro and in tumor xenografts. This finding is clinically relevant, as we identified a subset of patients with prostate cancer and homozygous *SHLD2* deletions, defining a patient population that could extensively benefit from Polθi combined with RT and offering a potential biomarker for patient stratification.

We found that ~20% of *PTEN*-null prostate tumors also harbor homozygous deletions in *SHLD2* and that most of *SHLD2*-null tumors are likewise *PTEN* null. The inverse correlation between the distance of neighboring genes from *PTEN* and their codeletion incidence suggests that *PTEN* is the tumor suppressor acting primarily as a cancer driver, while the deletion in the nearby genes—including *SHLD2*—are likely passenger deletion events. *PTEN* loss has been correlated with adverse outcomes in patients with prostate cancer, including those treated with RT (*21*, *41*–*44*). We also demonstrate that *PTEN* deficiency does not compromise the enhanced radiosensitizing effect of Polθi observed upon *SHLD2* loss. Therefore, SHLD2 loss appears to represent a collateral vulnerability to Polθi following RT in PTEN-deficient tumors, offering a strategy to effectively target a subset of this group of patients (*12*, *45*).

Of note, we also demonstrated that *SHLD2* loss sensitizes cancer cells to RT alone both in vitro and in vivo. This finding raises the possibility that patients with *PTEN*-deficient/*SHLD2*-deficient tumors may experience better outcomes after RT than those with *PTEN*-deficient/*SHLD2*-proficient tumors. However, we were unable to test this hypothesis due to the limited number of RT-treated patients with available clinical outcome data in the publicly available cohorts analyzed, the prolonged interval between tumor sampling and RT in many patients—creating uncertainty as to how accurately the samples reflect SHLD2 status at the time of treatment—as well as the substantial heterogeneity in the type, combination, and scheduling of additional therapies which may confound interpretation of RT-specific outcomes. Future studies should address how *SHLD2* loss influences prognosis both in *PTEN*-proficient and *PTEN*-deficient patient populations, to better gauge the potential clinical impact of combining Polθi with RT in these subgroups.

Genetic or pharmacological inhibition of Polθ previously revealed a synthetically lethal interaction between Polθ and components of the HR repair pathway, such as *FANCD2*, *BRCA1*, or *BRCA2* (*8*–*10*). Depletion of components of the TP53BP1/Shieldin pathway has also been shown to sensitize cancer cells to Polθi monotherapy specifically in HR-deficient backgrounds (*10*). In contrast, the *SHLD2^−/−^* models used in our study are HR proficient, underscoring the potential utility of Polθi beyond HR-deficient tumors. In line with our earlier reports (*2*), the present study confirms that the addition of Polθi to fractionated RT is well tolerated in vivo. Notably, several Polθ inhibitors—including the class of compounds tested in this study—have now entered early-phase clinical trials (NCT05898399, NCT04991480, NCT06077877, NCT05687110, NCT06666270, NCT06545942, and NCT06560632), further supporting the translational promise of this combination strategy.

Among the other hits whose loss conferred enhanced sensitization to the combination of Polθi and RT, we identified several genes involved in the major DSB repair pathways, namely, HR and NHEJ. This is in agreement with the role of Polθ-mediated repair of DSBs as a backup mechanism to HR and NHEJ (*8*, *9*, *13*, *14*) and supports the notion that increased radiation sensitivity may be associated with enhanced Polθi-mediated radiopotentiation, as observed in the context of *SHLD2* loss. Although loss or inactivation of some of these genes like *ERCC6L2* (*46*), *PRKDC* (*DNA-PK*) (*47*, *48*), and *ATM* (*49*) has been shown to sensitize cells to RT alone, this report identifies them as potential modulators of Polθi-induced radiosensitization. Further studies are warranted to validate these findings and evaluate their relevance for patient selection. It is tempting to speculate that available inhibitors targeting some of these proteins—such as ATM or DNA-PK—could potentiate the radiosensitizing effect of Polθi. However, clinical translation of these strategies would have to face the complexity of triple combination therapies and the toxicity of these inhibitors when combined with RT (*50*, *51*).

Within the TP53BP1/Shieldin axis, *TP53BP1* and *SHLD3* emerged as hits in the RT and Polθi arm of the screen, in addition to *SHLD2*. However, follow-up colony formation assays evidenced only a role for *TP53BP1* in modulating the radiosensitizing effect of Polθi, whereas *SHLD3* targeting did not enhance Polθi-mediated radiosensitization in this setting. Conversely, although *MAD2L2* did not emerge as a hit in the RT and Polθi arm of the screen, its targeting in validation assays phenocopied *SHLD2* loss, supporting a functional contribution to Polθi-mediated radiosensitization. Collectively, these findings suggest differential functional contributions of individual TP53BP1/Shieldin components in the context of Polθi following RT. The differences between the pooled screen and orthogonal validation assays likely reflect screening artifacts and/or differences in screening sensitivity and experimental context, such as radiation regimen (fractionated versus single dose, potentially revealing cell cycle dependencies), or cell line–specific genetic context. While pooled CRISPR screens are powerful discovery tools, functional validation in dedicated assays remains essential to define biologically relevant interactions. The performance metrics of our screen—including library representation and appropriate behavior of positive and negative controls—support its overall robustness.

The TP53BP1/Shieldin pathway has been implicated in limiting DNA resection by promoting fill-in synthesis at ssDNA overhangs formed after short-range resection by the MRE11/CtIP complex (*4*, *31*, *33*, *34*). SHLD2 provides Shieldin with the ability to bind to ssDNA, a function central to its role in opposing DNA end resection (*52*). While the TP53BP1/Shieldin pathway is thought to promote NHEJ and suppress HR through this antagonistic action on resection (*4*, *27*, *29*–*34*), its role in modulating Polθ-mediated repair remains obscure. Our study suggests that *SHLD2* loss increases the reliance on Polθ following RT, with Polθ activity limiting DSB accumulation and chromosomal instability. This provides a mechanistic basis for the enhanced radiosensitization observed upon Polθi in *SHLD2*-deficient cells.

Since the ssDNA overhangs resulting from short-range resection constitute optimal substrates for MMEJ (*4*,*35*–*38*), we hypothesized that *SHLD2* loss may increase the pool of resected ends available for Polθ-mediated MMEJ, thereby making cells more dependent on Polθ to survive RT. However, we show that depletion of the short-range resection factors MRE11 or CtIP—conditions known to markedly inhibit MMEJ (*36*–*38*, *40*)—does not suppress Polθi-induced radiosensitization in *SHLD2*-deficient cells, despite significant inhibition of resection, particularly upon CtIP knockdown. Consistently, we demonstrate that depletion of these short-range resection factors does not reverse the accumulation of DSBs induced by Polθi following RT in *SHLD2*-deficient cells. Therefore, our findings suggest that Polθ promotes the survival of *SHLD2*-deficient cells after RT exposure via a pathway independent of MRE11/CtIP-mediated DNA end resection.

Our results show that there is a residual recruitment of the HR factor RAD51 to DSB foci upon depletion of either CtIP or MRE11, which may indicate that a certain level of DNA end resection still occurs independently of these short-range resection factors, potentially allowing for some level of Polθ-mediated MMEJ. Alternatively, recent studies have shown that Polθ also functions in other pathways distinct from DSB repair by MMEJ. Specifically, Polθ has been implicated in preventing the accumulation of ssDNA gaps that emerge spontaneously behind the replication fork in HR-deficient cells and which can subsequently be transformed into more cytotoxic DSBs (*53*–*55*). Given that both Polθ and TP53BP1/Shieldin are implicated in fill-in DNA synthesis, it is conceivable that they may act redundantly to ensure gap filling and prevent ssDNA accumulation following RT. *SHLD2* loss has been reported to potentiate the spontaneous accumulation of postreplicative ssDNA gaps induced by Polθi in HR-deficient cells (*53*). Therefore, we propose that *SHLD2* deficiency may increase the pool of RT-induced DNA damage substrates that are amenable to Polθ-mediated repair, potentially encompassing noncanonical resection-dependent substrates and/or resection-independent intermediates. Further studies are needed to fully elucidate the mechanism underlying the novel synthetically lethal interaction between *SHLD2* and Polθ following RT revealed in the present study.

In summary, we demonstrate that *SHLD2* loss increases the susceptibility of cancer cells to the radiosensitizing effect of Polθi. *SHLD2* loss may serve as a biomarker for selecting patients with prostate cancer most likely to benefit from Polθi in combination with RT. This vulnerability reflects an increased dependence of *SHLD2*-deficient cells on Polθ, which, in this context, prevents DSB accumulation and maintains chromosomal stability, via a mechanism independent of MRE11/CtIP-mediated end resection. These findings support the clinical investigation of this combination strategy for patients with *SHLD2*-deficient tumors.

## MATERIALS AND METHODS

### Cell culture and treatments

Cell lines were cultured in a humidified incubator at 37°C and 5% CO_2_, passaged before reaching 80% confluency, and were routinely tested for mycoplasma using the MycoAlert PLUS Mycoplasma Detection kit (Lonza). Details of cell lines, their source, and cell culture media are provided in table S1. ART558 was produced as described previously (*10*). ART899 was produced from its nondeuterated form, ART812, with the addition of deuterium during synthesis, as previously described (*2*). Compounds were stored in powder form in a vacuum chamber at room temperature in the dark. Compounds were reconstituted in dimethyl sulfoxide (DMSO) at 12 mM, and these stocks were kept at room temperature in the dark. ART558 was added 1 hour before RT.

### Cell line panel experiment

We used a panel comprising 16 lung, 20 colorectal, and 18 head and neck cancer cell lines (listed in fig. S1 and table S1). Cells were seeded in 24-well plates at optimized seeding densities. The day after seeding, cells were treated with 3 μM ART558 and exposed to the first radiation fraction of a 3 × 2 Gy regimen; subsequent fractions were delivered every 24 hours. The inhibitor was maintained throughout the experiment. Cells were incubated for 14 days posttreatment and then stained with crystal violet. Cell viability in the cell line panel was quantified using crystal violet solubilization, since many of the cell lines used are not capable to grow detectable colonies. Stained samples were incubated for at least 30 min with 10% acetic acid to solubilize the crystal violet dye. After complete solubilization, the optical density at 590 nm of the acetic acid solution was measured using a Clariostar plate reader. Absorbance values were then normalized to the seeding numbers, and surviving fractions (SF) were calculated as the corrected absorbance of the irradiated condition divided by that of the unirradiated control. The extent of radiosensitization by Polθi was estimated as SF_ART558_/SF_DMSO_. The SF_ART558_/SF_DMSO_ values were then correlated with POLQ expression levels and the frequency of exomic Polθ-generated scars. POLQ expression values were retrieved from the DepMap Public 24Q4 RNA sequencing dataset (*56*). To quantify the frequency of exomic Polθ-generated scars—defined here as deletions of ≥5 bp flanked by microhomologies of ≥2 bp—we applied the computational pipeline described by Carvajal-Garcia *et al.* (*7*) to the DepMap 24Q4 whole-exome sequencing dataset (*56*). Scar frequencies were normalized for each cell line to their total exonic length in base pairs, calculated as the sum of Ensembl-annotated exon lengths weighted by the copy number at the gene level retrieved from DepMap.

### CRISPR KO screen

We generated a Cas9-expressing cell line (DLD-1 Cas9) by infecting DLD-1 cells with LVCAS9BST-1EA lentiviral particles carrying a vector encoding for blasticidin resistance and Cas9 genes (*57*). After infection, cells were treated with blasticidin (10 μg/ml) for 10 days. Optimal (>95%) Cas9 cutting efficiency in Cas9-expressing DLD-1 cells was demonstrated using the CRISPRuTest (Cellecta) (fig. S2, B and C).

For the CRISPR KO screen, we utilized a pooled lentiviral CRISPR KO library targeting genes implicated in the DNA damage response and in cancer pathogenesis (data S1A). This library comprised 16,732 guide RNAs targeting 2776 genes, with approximately six sgRNAs per gene, along with 100 nontargeting control sgRNAs. Fifty million DLD-1 Cas9 cells were infected with this library at a multiplicity of infection of 0.4 and incubated for 7 days with puromycin (2 μg/ml) to select for cells with successful donor sequence integration.

The experimental design and assay timeline are outlined in fig. S2A. Library-infected cells were seeded 1 day before treatment, with triplicates per treatment group. A minimum of 13 million library-infected cells were used for each replicate and cultured throughout the screen to maintain a library representation of at least 760-fold. Treatment groups were as follows: DMSO (vehicle control), 6 μM ART558, 0.5, 1, 0.5 Gy + 6 μM ART558, and 1 Gy + 6 μM ART558. For RT treatment, cells received four fractions of 0.5 or 1 Gy every 24 hours using a CellRad (Faxitron) x-ray irradiator at a dose rate of 1.02 Gy/min. ART558 was added 1 hour before the first RT dose.

On day 4, cells were detached 3 hours post-RT, counted, and reseeded to maintain exponential cell growth and library representation. At this point, 6 μM ART558 was added again and kept in the media until collection of cells. Cell pellets were collected for DNA extraction on days 8, 11, and 15 after treatment initiation. The growth curves of cells exposed to the different treatments over the course of the experiment are displayed in fig. S2D.

Genomic DNA (gDNA) extraction and purification were carried out using the GenElute Mammalian Genomic DNA Miniprep Kit (G1N70-1KT, Sigma-Aldrich), and gDNA solutions were prepared at a concentration of 100 ng/μl for polymerase chain reaction (PCR). sgRNA sequences integrated in the gDNA were recovered by nested PCR amplification utilizing vector backbone–directed primers. Parallel PCRs were performed with a Veriti 96-well thermal cycler (Applied Biosystems) under the following conditions: PCR1: 95°C for 10 s, 38 cycles of 95°C for 10 s, 55°C for 10 s, 72°C for 20 s, and 72°C for 1 min; PCR2: 95°C for 10 s, 12 cycles of 95°C for 10 s, 56°C for 10 s, 72°C for 20 s, and 72°C for 1 min; PCR3: 95°C for 10 s, 12 cycles of 95°C for 10 s, 50°C for 10 s, 72°C for 22 s, and 72°C for 1 min.

PCR products from parallel reactions were subsequently pooled and purified using the GenElute PCR Clean-Up Kit (NA1020-1KT, Sigma-Aldrich). Sequencing reactions were performed on an Illumina NextSeq 500/550 using the High Output v2 kit, following the manufacturer’s instructions. The sequencing reads were computationally demultiplexed and trimmed to remove barcodes and adapters. The trimmed reads were aligned to the reference genome with BowTie2 (*58*), filtering out ambiguous hits. SAMtools (*59*) was then applied to process the alignment files and generate depth data for the library sequences. *z*-Scores for each sequence was calculated to provide a normalized dataset.

The sgRNA counts obtained as described above were analyzed with MAGeCK (*60*) to generate gene-level enrichment scores for each of the treatment groups in relation to the corresponding control group (data S1C). Screen quality was assessed at both sgRNA and gene levels. The receiver operating characteristic curve was generated using “AchillesCommonEssentialControls.csv” and “AchillesNonessentialControls.csv” from DepMap version 22Q4 and used to define essential and nonessential genes, respectively. This analysis showed high specificity and sensitivity of essential genes, utilized here as positive controls (fig. S2E).

A negative sgRNA enrichment for a given gene in the ART558, RT, or ART558 + RT groups was considered significant when that gene exhibited a MAGeCK negative selection score (neg.score) ≤ 0.001 and a negative selection *P* value (neg.p.value) < 0.01 in at least two conditions within those groups—i.e., across the three collection time points and/or, for RT-treated samples, across the two radiation regimens. Genes meeting these thresholds were ranked by the sum of the log fold changes (∑neg.logFC) of all qualifying conditions (i.e., those time points or radiation regimens in which neg.score ≤ 0.001 and neg.p-value <0.01). This yielded a list of the candidate genes whose KO was associated with enhanced sensitivity to either ART558 or RT (data S1D).

We then generated a list of genes whose KO was associated with enhanced radiosensitization by Polθi, with an effect above the expected additivity of the two single treatments, calculated using the Bliss independence model (*61*). To this aim, we first selected the hits fulfilling the above mentioned criteria of neg.score ≤ 0.001 and neg.p.value < 0.01 for ART558 + RT. Then, we determined the expected combined neg.lfc (*E*_RT+ART558_) by summing the individual observed (*O*) effects of RT and ART558 alone according to the following equation: *E*_RT+ART558_ = *O*_RT_(neg.lfc) + *O*_ART558_(neg.lfc). Then, the deviation between the observed neg.lfc values for the combination treatment (*O*_RT+ART558_) and *E*_RT+ART558_ was calculated as: Δ*O* − *E* = *O*_RT+ART558_ − *E*_RT+ART558_. Last, a specific gene KO was classified as a genetic vulnerability to the ART558 + RT combination when Δ*O* − *E* ≤ −0.1 in at least two of the six ART558 + RT treatment conditions. For each gene, the neg.lfc scores across the qualifying treatment conditions (Δ*O* − *E* ≤ −0.1) were summed to yield an overall ∑(Δ*O* − *E*) score. Genes were then ranked by descending ∑(Δ*O* − *E*) (Fig. 2D and data S1E).

### Protein interaction network analysis

STRING portal (https://string-db.org/) was used to generate the protein-protein interaction network of the candidate modulators of Polθi-mediated radiosensitization identified in the CRISPR KO screen.

### Interrogation of cBioportal for frequency of *SHLD2* loss in clinical samples

A nonredundant set of prostate studies with gene copy number data for more than 100 patient samples was selected on cBioPortal and queried for *SHLD2* homozygous deletions (SHLD2: HOMDEL). For the two representative studies TCGA (PanCancer Atlas) (*19*, *20*) and SU2C/PCF (*17*) Dream Team that provided data amenable for codeletion representation, homozygous deletions were plotted for the main genes in the genomic region between *PTEN* and *SHLD2* to assess the frequency and co-occurrence pattern of homozygous deletions (deep deletions defined as per cBioportal). The results pertaining to the TCGA (PanCancer Atlas) (*19*, *20*) are based on data generated by the TCGA Research Network: https://cancer.gov/tcga.

### Colony formation assays

Cells were seeded in either 24-well or 6-well plates and allowed to settle for 4 to 5 hours. ART558 (4 μM) was added to each well with DMSO utilized as a negative control. An x-ray irradiator (CellRad by Faxitron; dose rate, 1.02 Gy/min) was used to irradiate colony formation assay (CFA) samples in Fig. 4. A Caesium-137 irradiator (GSR D1 from Gamma Service; dose rate, 1.2 Gy/min) was used to irradiate CFAs in Fig. 5 and figs. S5 and S6. For CFAs in Fig. 4, media containing ART558 or DMSO was left for the duration of the assay. For CFAs in Fig. 5, media containing ART558 or DMSO was replaced with fresh media 3 days after RT.

The plates were allowed to form colonies and then stained and fixed with crystal violet 12 to 14 days later. Colonies were analyzed using an Oxford Optronics Gel Count machine and GelCount software. The plating efficiency (PE) was defined as: PE = average colony number/cells plated. In the experiments testing the single-agent effect of ART558 (Fig. 4, D to F), the SF relative to the DMSO control was calculated as PE_ART558_/PE_DMSO._ In the experiments involving RT treatment, the SF relative to the unirradiated control (no RT) was calculated as PE_RT_/PE_No RT_. To estimate the extent of radiosensitization by ART558, we calculated the ratio of the SFs of ART558-treated to DMSO-treated cells.

To assess the synergy between RT and Polθi in vitro, we applied a Bliss independence model based on the PE data from the CFA. For each independent repeat, we calculated the expected PE for the combination under an additivity assumption as the sum of the PE of the single treatments: PE_ART558/RT_ = PE_ART558_ + PE_RT_. The statistical significance of the synergistic interaction was assessed by comparing the observed PE for the combination treatment with the expected PE under the additivity assumption (PE_ART558/RT_) using a one-sample *t* test. A combined *P* value for each replicate was calculated using the Fisher’s test. The magnitude of the interaction was estimated as the difference between the observed and expected plating efficiencies (Δ*O* − *E*), with a Δ*O* − *E* < 0 indicating synergy.

### Immunofluorescence microscopy

Cells were seeded in black-wall 96-well plates and left to attach overnight. The following day, cells were treated with 4 μM ART558. An x-ray irradiator (CellRad by Faxitron; dose rate, 1.02 Gy/min) was used to irradiate the plates. At the corresponding time points after treatment, cells were washed with phosphate-buffered saline (PBS) and preextracted with CSK buffer (table S2) for 6 min. Cells were then fixed with 4% paraformaldehyde in PBS for 10 min. Samples were incubated for 1 hour at room temperature with blocking buffer [0.5% bovine serum albumin (BSA) and 0.5% Triton in PBS], then with primary antibodies (see table S3) diluted in blocking buffer overnight at 4°C, and then with the secondary fluorescent antibodies (see table S3) for 1 hour at room temperature in the dark. Samples were imaged using an Operetta CLS system and processed and analyzed on the Harmony image analysis software. An analysis pipeline was generated and applied to all acquired images in the Harmony software to quantify foci and micronuclei.

### Generation of *SHLD2*-deficient clones, KO confirmation, and *SHLD2* restoration

The *SHLD2^−/−^* CAL-51 clones E1 and E2, DU145 clone A2, and 22Rv1 clones A6, C1, and G6 were generated by Oxford Genetics (Oxford, UK), using CRISPR-Cas9 editing as described previously (*10*). Briefly, sgRNA for CRISPR-Cas9 were designed to target the *SHLD2* gene (reference gene ENSG00000122376). The 5′ → 3′ sequence of the genomic sgRNA target used was ATTACAGCATCTCAGAAGAT. Pools of cells carrying the edited gene were generated by transient cotransfection of the sgRNA complexed with CRISPR-Cas9 protein. Single cells were isolated, and the targeted exon was sequenced by Sanger sequencing. Selected clones with out-of-frame insertion/deletions in all alleles were expanded and validated by PCR followed by high-throughput sequencing, which confirmed that all clones used in the study carried out-of-frame insertions or deletions in all *SHLD2* alleles (fig. S4A). During the course of the experiments, representative clones were further checked by PCR, Sanger sequencing, and Inference of CRISPR Edits (ICE) analysis to validate that the *SHLD2* KO status was maintained in vitro and in vivo (figs. S4 and S7). More methodological details on the KO confirmation are provided in Supplementary Methods. To restore *SHLD2* expression in 22Rv1 *SHLD2^−/−^* clones, cells were infected with a lentiviral vector carrying either the full open reading frame (ORF) of *SHLD2* or the control ORF of GFP (Ex-A8388-Lv105 and Ex-EGFP-Lv105 from Labomics, respectively), as previously described (*10*). Infected cells underwent puromycin selection and expansion, and *SHLD2* restoration was confirmed by Western blotting (fig. S4D).

### Reverse siRNA transfection

siRNA transfections were performed using RNAiMax (Invitrogen) as the transfection reagent, in a reverse transfection procedure following the manufacturer’s instructions. The sense strand sequences of the siRNAs (Silencer Select, Thermo Fisher Scientific) are provided in table S5. siRNAs were used at a concentration of 40 nM. Treatments were started 72 hours after transfection. Knockdown was confirmed by Western blotting in cell lysates collected at the time of RT.

### Western blotting

For detailed reagent information, see table S6. Cells were washed in PBS and kept at −80°C until lysate preparation. Cells were lysed with radioimmunoprecipitation assay buffer (Pierce) supplemented with protease (Merck) and phosphatase (Sigma-Aldrich) inhibitors. Protein concentration was quantified using the bicinchoninic acid (BCA) assay (Thermo Fisher Scientific). Equal protein amounts (25 to 50 μg) were resolved by SDS–polyacrylamide gel electrophoresis in 4 to 20% or 7.5% tris-glycine gels (Bio-Rad) and transferred to nitrocellulose membranes using a Transblot turbo transfer system (Bio-Rad). Membranes were blocked in either 3% BSA or 5% milk dissolved in tris buffer saline/0.01% Tween 20 (TBST), which were also used for subsequent incubation steps. Membranes were probed overnight at 4°C with primary antibodies. The membranes were then washed with TBST and incubated with the corresponding horseradish peroxidase–conjugated secondary antibody. After washing twice in TBST, membranes were incubated with ECL prime (Cytiva) before imaging on a Chemi-Doc imager (Bio-Rad). See table S7 for the specific primary and secondary antibodies used.

### In vivo experiments

The project license covering the animal work was approved by the Animal Welfare and Ethical Review Body at the University of Oxford and granted by the UK Home Office Animals in Science Regulation Unit under the Animals (Scientific Procedures) Act 1986. We used male NOD.Cg-Rag1^tm1Mom^ Il2^rgtm1Wjl^/SzJ (NRG) mice, aged 7 to 8 weeks. Mice were subcutaneously injected in the right flank with 2.5 × 10^6^ DU145 *SHLD2^WT^* or *SHLD2^−/−^* clone A2 cells suspended in a 1:1 mixture of Matrigel and PBS, in a total volume of 100 μl. Following tumor cell injection, mice were randomly assigned via a random number generator (random.org) to one of four treatment groups: vehicle control, ART899, RT, or ART899 combined with RT. The vehicle consisted of 5% DMSO, 5% ethanol, 20% d-α-tocopheryl polyethylene glycol 1000 succinate (TPGS), and 30% polyethylene glycol 400 and diluted in ultrapure water. Mice received either vehicle alone or ART899 (150 mg/kg body weight) dissolved in vehicle, administered by oral gavage twice daily for 14 days (days 0 to 13), at a volume of 0.043 ml per 10 g body weight. The first daily gavage dose was given 1 to 2 hours before RT, and the second dose was administered 6 hours after the first dose. RT was delivered to tumors using a Gulmay 320 x-ray generator (Gulmay Medical Ltd., Camberley, UK) at 300 kV and 10 mA. The fractionated RT regimen consisted of four consecutive doses of 2 Gy delivered every 24 hours, on days 0 to 3, under isoflurane anesthesia. During irradiation, mice were covered with a lead shield that exposed only the tumor to the x-rays.

Treatments started when tumors reached an approximate size of 160 ± 4 mm^3^ (mean ± SD). Animals were weighed before gavage dosing during treatment and twice weekly thereafter. Tumor size was assessed at least two times a week with calipers and calculated according to the formula: (length × width^2^)/2. Mice were euthanized with pentobarbital when tumors reached 1000 mm^3^. In the graphs showing tumor size versus days after treatment start, curves were represented up to the time point where the first mouse in the corresponding group reached this tumor size threshold. Survival fractions at specific time points after treatment initiation were estimated on the basis of the number of mice that reached a tumor size of 1000 mm^3^ relative to the initial group size, using the Kaplan-Meier method. Statistical significance of the difference between survival curves was assessed using the log-rank (Mantel-Cox) test.

Synergy between ART558 and RT in the in vivo experiments was assessed using the Bliss independence model applied to survival analysis, as previously described (*61*). A survival curve representing the expected effect of the combined treatments under the assumption of independent action was generated using the formula *S*_ART899/RT_ = 1 − [1 − *S*_ART899_ (*t*)] × [1 − *S*_RT_ (*t*)], where *S* denotes the survival as a function of time (*t*). The statistical significance of the difference between the observed survival of the combination treatment and the modeled survival *S*_ART899/RT_ was then evaluated using the log-rank (Mantel-Cox). The magnitude of the interaction was quantified by the hazard ratio between the combination treatment and the Bliss-predicted model. Synergy was defined as a significantly greater observed survival of the combination treatment compared with the Bliss-predicted survival, with a hazard ratio > 1 indicating a synergistic interaction.

### Statistical analysis

The specific statistical analyses are indicated in the figure legends and, where relevant, detailed in the corresponding Materials and Methods section. All graphs were generated, and statistical analyses were performed with GraphPad Prism, unless otherwise specified.
