# Supplementary material for: SHLD2 loss is a synthetic vulnerability to Polθ inhibition combined with radiotherapy
Source: Sci Adv. 2026 Jun 12;12(24):eaeb4508. doi: 10.1126/sciadv.aeb4508 (PMC13262640; doi:10.1126/sciadv.aeb4508)
Supplement: Supplementary file 1 — Supplementary Methods Tables S1 to S7 Legend for data S1 Figs. S1 to S7 [file sciadv.aeb4508_sm.pdf]

Science Advances
AAAS

Supplementary Materials for

SHLD2 loss is a synthetic vulnerability to Polθ inhibition combined with radiotherapy

Gonzalo Rodriguez-Berriguete *et al.*

Corresponding author: Marco Ranzani, drmarcoranzani@gmail.com;
Geoff S. Higgins, geoffrey.higgins@oncology.ox.ac.uk

Sci. Adv. **12**, eaeb4508 (2026)
DOI: 10.1126/sciadv.aeb4508

The PDF file includes:

Supplementary Methods
Tables S1 to S7
Legend for data S1
Figs. S1 to S7

Other Supplementary Material for this manuscript includes the following:

Data S1

Supplementary Methods

SHLD2 knockout confirmation

The *SHLD2* PCR forward primer was CAAGGAGAGAGGACATGTTAGC (Sigma Aldrich SY230122662-075) and *SHLD2* PCR reverse primer was AGGGTTTACAGACTAATTTTTCCAG (Sigma Aldrich SY230122662-076). Genomic DNA was extracted using Lucigen QuickExtract™ DNA Extraction Solution (QE09050) and subjected to 65°C for 10 minutes, followed by 98°C for 5 minutes using a MasterCycler X50S. PCR was performed in duplicate samples using Q5 Hot Start High-Fidelity 2X Master Mix (New England Biolabs M0494S) in a Mastercycler X50 thermocycler. PCR cycling conditions were as follows: initial denaturation at 98°C for 30 seconds; 30 cycles of denaturation at 98°C for 10 secs; annealing at 63°C for 30 seconds; and extension at 72°C for 30 secs; final extension step at 72°C for 2 mins (see Supplementary Table 4). The PCR product was sequenced by Source Bioscience and the sequencing data was analysed using the ICE platform (Synthego, https://ice.editco.bio/#/) to obtain the indel frequency spectrum and determine the genetic make-up of the KO clones.

Cell cycle analysis

Suspended and adherent cells were collected together and fixed in ice-cold 70% ethanol. After fixation, cells were washed with PBS and incubated with propidium iodide (PI; 50 µg/mL) and RNase A (200 µg/mL) in PBS for 20 min. Samples were then analysed by flow cytometry using FlowJo (BD). Doublets were excluded from the analysis. The proportions of cells in G1, S, and G2/M phases were determined based on PI fluorescence intensity, which reflects cellular DNA content.

Supplementary Table 1. Cell lines, origin and culture media.

Cell line ID	Source	Media + supplements
DU 145	ATCC (HTB-81)	EMEM + 10% FBS
CAL-51	DSMZ (ACC 302)	DMEM (high glucose) + 10% FBS
22Rv1	ATCC (CRL-2505)	RPMI 1640 + 10% FBS
Calu-6	ATCC (HTB-56)	EMEM + 10% FBS
A549	ATCC (CCL-185)	Ham's F-12K (Kaighn's) + 10% FBS
NCI-H2073	ATCC (CRL-5918)	RPMI 1640 + 10% FBS
NCI-H1651	ATCC (CRL-5884)	DMEM:F12 + 0.02 mg/mL insulin, 0.01 mg/mL transferrin, 25 nM sodium selenite, 50 nM Hydrocortisone, 1 ng/mL EGF, 0.01 mM ethanolamine, 0.01 mM phosphorylethanolamine, 100 pM triidothyronine, 0.5% BSA, 10 mM HEPES, 0.5 mM sodium pyruvate, 2 mM L-glutamine (final conc. 4.5 mM), 10% FBS
YD-15	Horizon Discovery Inc.	RPMI + 10% FBS + 25 mM HEPES + 25 mM Sodium Bicarbonate
HT-115	Horizon Discovery Inc.	DMEM + 15% FBS + 2 mM Glutamine
YD-8	Horizon Discovery Inc.	RPMI + 10% FBS + 25 mM HEPES + 25 mM Sodium Bicarbonate
COLO-320	Horizon Discovery Inc.	RPMI 1640 + 10% FBS
BICR 16	Horizon Discovery Inc.	DMEM + 10% FBS + 0.4 μg/mL Hydrocortisone
HCT-15	Horizon Discovery Inc.	RPMI 1640 + 10% FBS
DLD-1	ATCC (CCL-221)	RPMI 1640 + 10% FBS
YD-10B	Horizon Discovery Inc.	RPMI + 10% FBS + 25 mM HEPES + 25 mM Sodium Bicarbonate
SK-LU-1	ATCC (HTB-57)	EMEM + 10% FBS
CAL-33	DSMZ (ACC447)	DMEM (high glucose) + 10% FBS
SW620	Horizon Discovery Inc.	RPMI 1640 + 10% FBS
NCI-H1568	ATCC (CRL-5876)	RPMI 1640 + 10% FBS
NCI-H1299	ATCC (CRL-5803)	RPMI 1640 + 10% FBS
NCI-H747	Horizon Discovery Inc.	RPMI 1640 + 10% FBS
SW900	ATCC (HTB-59)	RPMI 1640 + 10% FBS
MDST8	Horizon Discovery Inc.	DMEM + 10% FBS
RCM-1	Horizon Discovery Inc.	RPMI:Ham’s F12 (1:1) + 10% FBS

Detroit562	Horizon Discovery Inc.	EMEM + 10% FBS
RKO	Horizon Discovery Inc.	EMEM + 10% FBS
HT29	Horizon Discovery Inc.	McCoy’s 5A + 10% FBS
SW48	Horizon Discovery Inc.	RPMI 1640 + 10% FBS
HCT-8	Horizon Discovery Inc.	RPMI + 10% Horse Serum
SW480	Horizon Discovery Inc.	RPMI 1640 + 10% FBS
PE/CA-PJ15	Horizon Discovery Inc.	IMDM + 10% FBS
MORCPR	ECACC (Sigma-Merck)	RPMI 1640 + 10% FBS + 1 µg/mL cisplatin
KM12	Horizon Discovery Inc.	RPMI 1640 + 10% FBS
SNU-1066	Horizon Discovery Inc.	ATCC-formulated RPMI + 10% FBS + 25 mM HEPES + 25 mM Sodium Bicarbonate
FaDu	Horizon Discovery Inc.	EMEM + 10% FBS
PE-CA-PJ41-cl D2	Horizon Discovery Inc.	IMDM + 10% FBS
YD-38	Horizon Discovery Inc.	RPMI + 10% FBS + 25 mM HEPES + 25 mM Sodium Bicarbonate
HSC-3	Horizon Discovery Inc.	EMEM + 10% FBS
A253	ATCC (HTB-41)	McCoy's 5A Medium + 10% FBS
LS-411N	Horizon Discovery Inc.	RPMI 1640 + 10% FBS
HSC-4	Horizon Discovery Inc.	EMEM + 10% FBS
BICR 56	Horizon Discovery Inc.	DMEM + 10% FBS + 0.4 µg/mL Hydrocortisone
NCI-H1650	ATCC (CRL-5883)	RPMI 1640 + 10% FBS
SW837	Horizon Discovery Inc.	RPMI 1640 + 10% FBS
WiDr	Horizon Discovery Inc.	EMEM + 10% FBS
LS-123	Horizon Discovery Inc.	EMEM + 10% FBS
SNU-1076	Horizon Discovery Inc.	ATCC-formulated RPMI + 10% FBS + 25 mM HEPES + 25 mM Sodium Bicarbonate
NCI-H292	ATCC (CRL-1848)	RPMI 1640 + 10% FBS
HT55	Horizon Discovery Inc.	EMEM + 20% FBS

HCC-15	DSMZ (ACC496)	RPMI 1640 + 10% FBS
NCI-H1395	ATCC (CRL-5868)	RPMI 1640 + 10% FBS
NCI-H1373	ATCC (CRL-5866)	RPMI 1640 + 10% FBS
CAL-27	Horizon Discovery Inc.	DMEM + 10% FBS
BICR 6	Horizon Discovery Inc.	DMEM + 10% FBS + 0.4 μg/mL Hydrocortisone
NCI-H1869	ATCC (CRL-5900)	DMEM:F12 + 0.02 mg/mL insulin, 0.01 mg/mL transferrin, 25 nM sodium selenite, 50 nM Hydrocortisone, 1 ng/mL EGF, 0.01 mM ethanolamine, 0.01 mM phosphorylethanolamine, 100 pM triidothyronine, 0.5% BSA, 10 mM HEPES, 0.5 mM sodium pyruvate, 2 mM L-glutamine (final conc. 4.5 mM), 10% FBS
NCI-H441	ATCC (HTB-174)	RPMI 1640 + 10% FBS
LoVo	Horizon Discovery Inc.	Ham’s F12K + 10% FBS

Supplementary Table 2. Reagents and concentrations used to produce CSK buffer for immunofluorescence microscopy.

Reagent	Supplier	Reference	Final concentration
PIPES pH6.8	Thermo Fisher	J61786.AE	10 mM
NaCl	Sigma Aldrich	S9888-500G	100 mM
Sucrose	Sigma Aldrich	S0389-500G	300 mM
MgCl2	Sigma Aldrich	208337-100G	1.5 mM
EDTA	Merck	324506	5 mM
Protease inhibitor	Merck	11873580001	1 tablet per 10 mL
Phosphatase inhibitor	Merck	4906837001	1 tablet per 10 mL
Triton-X100	Merck	X100-100 mL	0.5%

Supplementary Table 3. Antibodies used for immunofluorescence microscopy.

Primary Antibodies	Species	Manufacturer; Product code	Dilution Factor	Blocking buffer
γH2AX	Mouse	Millipore; 05-636	1:2,000	0.5% BSA, 0.5% Triton
53BP1	Rabbit	Bethyl; A300-272A	1:2,000	0.5% BSA, 0.5% Triton
pATM	Mouse	Millipore; 10h11.e12	1:5,000	0.5% BSA, 0.5% Triton
RAD51	Rabbit	Millipore; ABE257	1:1,500	0.5% BSA, 0.5% Triton
Fluorescent Secondary Antibodies	Species	Manufacturer; Product code	Dilution Factor	Blocking buffer
DAPI	n/a	Sigma; D9542-5MG	1:5000	0.5% BSA, 0.5% Triton
AlexaFluor 488	Anti-Mouse	Thermo; A-11001	1:2,000	0.5% BSA, 0.5% Triton
AlexaFluor 568	Anti-Rabbit	Thermo; A-21245	1:2,000	0.5% BSA, 0.5% Triton

Supplementary Table 4. Thermocycler conditions for *SHLD2* primer set PCR.

Step	Temperature	Time
Initial Denaturation	98°C	30 seconds
25-35 cycles	98°C	10 seconds
	63°C	30 seconds
	72°C	30 seconds
Final Extension	72°C	2 minutes
Hold	4-10°C	

Supplementary Table 5. List of siRNAs.

siRNA	Product code	Manufacturer	ID	Sense strand sequence
Non-targeting Control (NT)	4390844	Thermo Fisher Silencer Select	n/a	n/a
MRE11 #1	4390824	Thermo Fisher Silencer Select	s8959	GAUAGACAUUAGUCCGGUUtt
MRE11 #2	4390824	Thermo Fisher Silencer Select	s8960	CCCGAAAUGUCACUACUAAtt
CtIP #1	4392420	Thermo Fisher Silencer Select	s11849	GUACAAGGUUUACAAGUAAtt
CtIP #2	4392420	Thermo Fisher Silencer Select	s11850	GGAUCUGUCUGAUCGAUUUtt
TP53BP1 #1	4392420	Thermo Fisher Silencer Select	s414313	GAAGGACGGAGUACUAAUAtt
TP53BP1 #2	4392420	Thermo Fisher Silencer Select	s414314	GCACAUUGUCACUCGUGUtt
RIF1 #1	4392420	Thermo Fisher Silencer Select	s30376	GGCCGAACACGGUAUCAAAtt
RIF1 #2	4392420	Thermo Fisher Silencer Select	s30377	GUGUUUAGGAGACUCGAAATtt
SHLD1 (C20orf196) #1	4392420	Thermo Fisher Silencer Select	s45349	CCAGACACCAGUAACUUAAtt
SHLD1 (C20orf196) #2	4392420	Thermo Fisher	s45350	CUGGAUAGAUUCUAUGAAAtt

		Silencer Select		
SHLD3 #1	HY-RS12875	MedChem Express	Set A-1	GUGAAACAGUACUUAACCAUU
SHLD3 #2	HY-RS12875	MedChem Express	Set A-2	GGAAAUCUGGGUAAACAAUCA
MAD2L2 (REV7) #1	4392420	Thermo Fisher Silencer Select	s20466	CAGACUCGCUGUUGUCUCAtt
MAD2L2 (REV7) #2	4392420	Thermo Fisher Silencer Select	s20467	ACCCGGAGCUGAAUCAGUAtt
POLQ	4392420	Thermo Fisher Silencer Select	s21059	CCGCUUUUGGAGUCAGUAAtt

Supplementary Table 6. List of reagents used for Western blotting.

Western blotting reagent	Manufacturer; product code
4-20% Mini-PROTEAN® TGX™ Precast Protein Gels (Tris-Glycine)	Bio-Rad; 4561094
7.5% Mini-PROTEAN® TGX™ Precast Protein Gels (Tris-Glycine)	Bio-Rad; 4561023
Trans-Blot Turbo Mini 0.2 µm Nitrocellulose Transfer Packs	Bio-Rad; 1704158
RIPA lysis buffer	Pierce; 89900
Protease inhibitor	Merck; 62650900
Phosphatase inhibitor	Sigma; P5726
ECL prime western blotting solution	Amersham Technologies; RPN 2236
BCA assay kit	Thermo Scientific; 23225

Supplementary Table 7. Antibodies used for Western blotting.

Primary Antibodies	Species	Manufacturer; Product code	Dilution Factor	Blocking buffer
CtIP	Mouse	Active Motif; 61141	1:500	3% BSA
MRE11	Rabbit	Abcam; ab10963	1:1,000	5% Milk
Polθ	Rabbit	Cell signalling; F8D8T	1:1,000	5% Milk
β-Actin	Mouse	Merck; A1978-100uL	1:10,000	5% Milk
**Secondary Antibodies**	**Species**	**Manufacturer; Product code**	**Dilution Factor**	**Blocking buffer**
Anti-Rabbit IgG HRP conjugated	Goat	Cell Signalling; 7074S	1:15,000	5% Milk
Anti-Mouse IgG HRP conjugated	Horse	Cell Signalling; 7076S	1:15,000	5% Milk

Supplementary Data 1

This is an excel file attached to this manuscript containing the data from the CRISPR KO screen.

Supplementary Figure 1 (accompanying Figure 1).

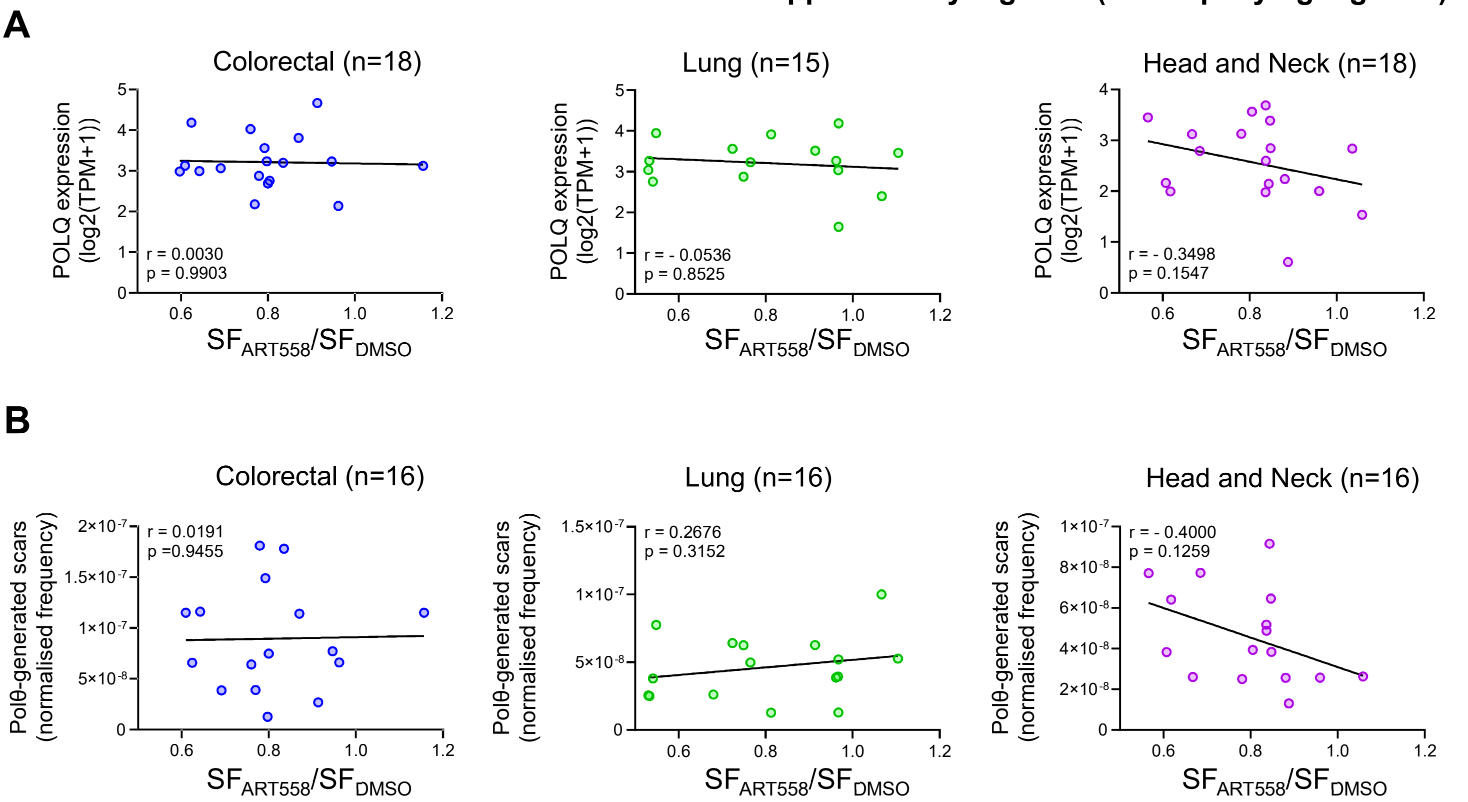

Supplementary Figure 1 (accompanying Figure 1).

(A) Correlation between *POLQ* expression and radiosensitisation by Polθi across tumour types.

(B) Correlation between the frequency of Polθ-generated scars and radiosensitisation by Polθi across tumour types.

Dots represent individual cell lines. Correlation coefficients (r) were calculated using the Spearman's method. The black lines are regression lines.

Supplementary Figure 2 (accompanying Figure 2).

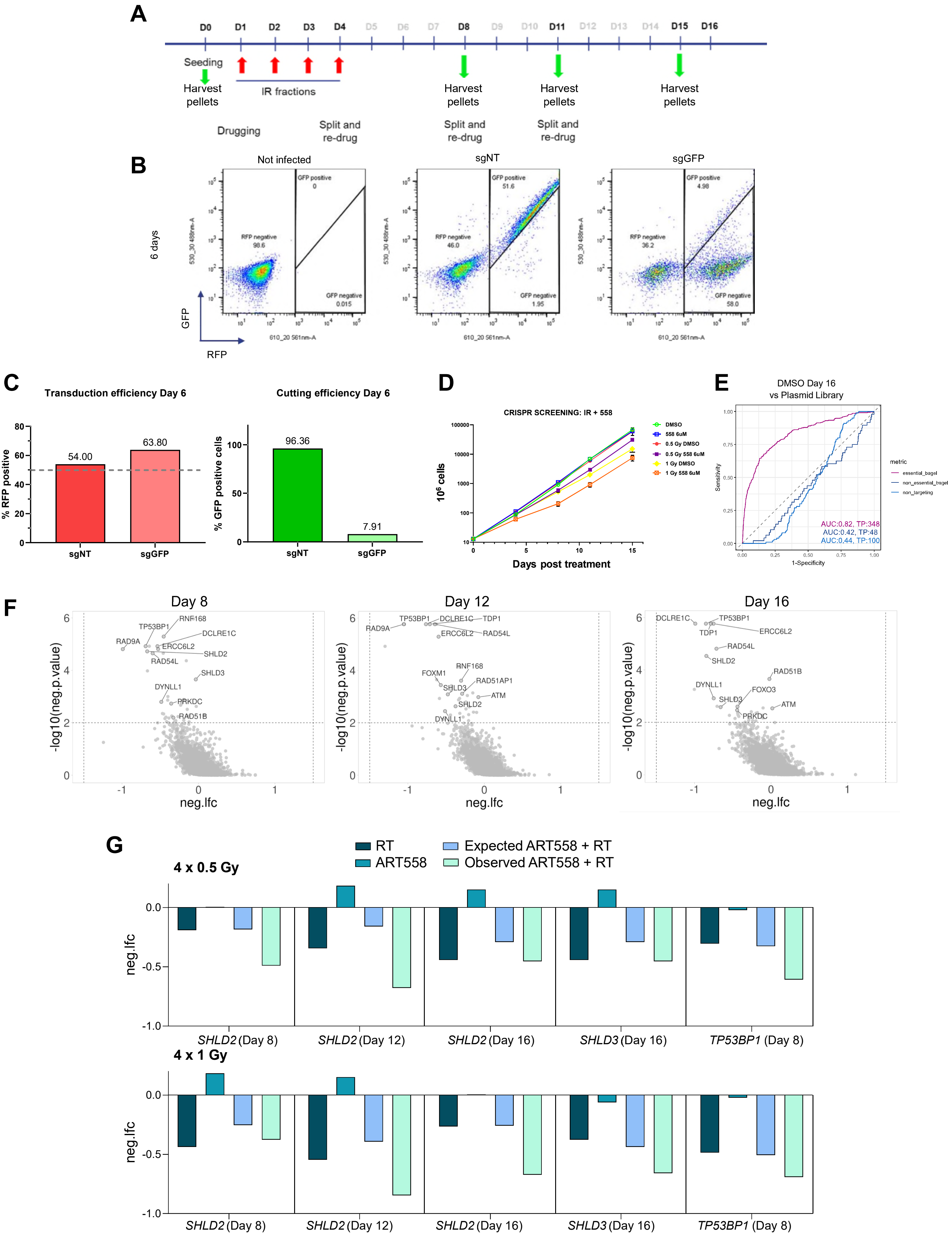

Supplementary Figure 2 (accompanying Figure 2).

(A) Experimental timeline of the CRISPR KO screen.

(B and C) Assessment of Cas9 cutting efficiency with CRISPRuTest^{TM}. Cas9-expressing DLD-1 cells were infected with a plasmid encoding for RFP and GFP, and either GFP-targeting sgRNA (sgGFP) or control sgRNA (sgNT). The higher the reduction in GFP signal analysed by FACS, the higher the cutting efficiency of Cas9.

(B) FACS plots showing Cas9 cutting efficiency. (C) Transduction efficiency and Cas9 cutting efficiency based on FACS plots shown in B.

(D) Growth curves of Cas9-expressing DLD-1 cells throughout the CRISPR screen.

(E) Receiver Operating Characteristic (ROC) curve showing sensitivity and specificity for essential and non-essential genes, and non-targeting sgRNAs for DMSO (day 16) vs plasmid library.

(F) Volcano plots highlighting the specific genes whose knockout synergised with combined Polθi (ART558) and RT (4 x 1 Gy) in at least two of the time points and/or RT schedules. neg.lfc: negative log fold change.

(G) Bar graphs representing the neg.lfc values relative to the untreated group for the Shieldin complex-related genes *SHLD2*, *SHLD3,* and *TP53BP1*. Only the timepoints and RT schedules where a meaningful deviation between the RT + ART558 (observed) and the sum of the RT alone and ART558 alone (expected) neg.lfc values (ΔObserved−Expected≤−0.1) for these genes are shown.

Supplementary Figure 3 (accompanying Figure 3).

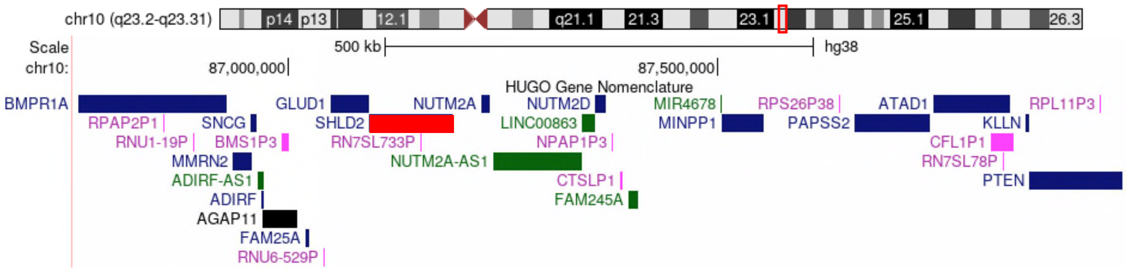

Supplementary Figure 3 (accompanying Figure 3).

UCSC Genome Browser visualisation of the genomic positions of *SHLD2* (red) and *PTEN* on chromosome 10 (GRCh38/hg38, Genome Reference Consortium).

Supplementary Figure 4 (accompanying Figure 4).

A

Cell line	Allele 1 (bp)	Allele 2 (bp)	Predicted frameshift (%)	R^2
CAL-51 clone E1	-17	-17	96	0.97
CAL-51 clone E2	-1	-28	98	0.94
DU145 clone A2	-1	-4	97	0.96
22Rv1 clone A6	+1	+8	96	0.94
22Rv1 clone C1	+1	-2	97	0.96
22Rv1 clone G6	+1	+1	97	0.98

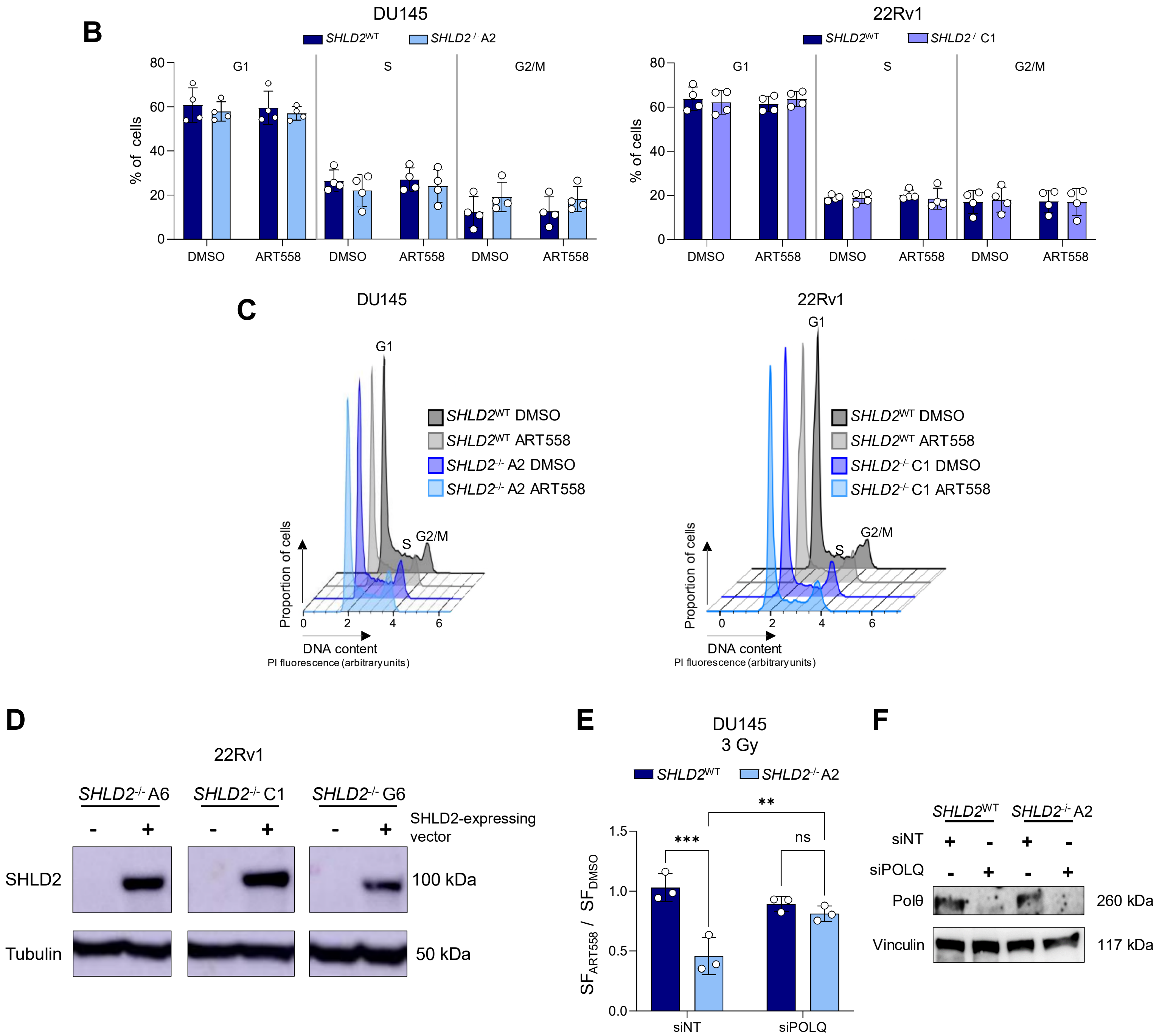

Supplementary Figure 4 (accompanying Figure 4).

(A) Confirmation of *SHLD2* KO status in the indicated cell lines. bp: base pair. (+) or (-) indicates an insertion or deletion, respectively.

(B) Cell-cycle phase distribution of DU145 cells (*SHLD2*^{WT} vs. *SHLD2*$^{-/-}$ clone A2) and 22Rv1 cells (*SHLD2*^{WT} vs *SHL2*$^{-/-}$ clone C1) treated with either DMSO or 4 µmol/L Polθi for 1 hour, assessed by flow cytometry analysis of DNA content following propidium iodide (PI) staining. No statistically significant differences were observed (two-way ANOVA with Tukey's post hoc test).

(C) Representative histograms corresponding to the data shown in (B).

(D) Western blot from lysates of *SHLD2*$^{-/-}$ 22Rv1 clones transduced with either a vector expressing *SHLD2* (+) or a GFP control vector (-).

(E) Extent of radiosensitisation by Polθi upon siRNA-mediated knockdown of Polθ in *SHLD2*^{WT} and *SHLD2*$^{-/-}$ A2 DU145 cells, assessed by colony formation assay.

(F) Western blots demonstrating depletion Polθ protein in DU145 cell lines from (E).

Data are representative of three independent experiments showing mean ± SD from triplicate wells. Statistical significance was calculated with two-way ANOVA and Tukey's post-hoc test. *p<0.05; **p<0.01; ***p< 0.001; ****p< 0.0001.

Supplementary Figure 5.

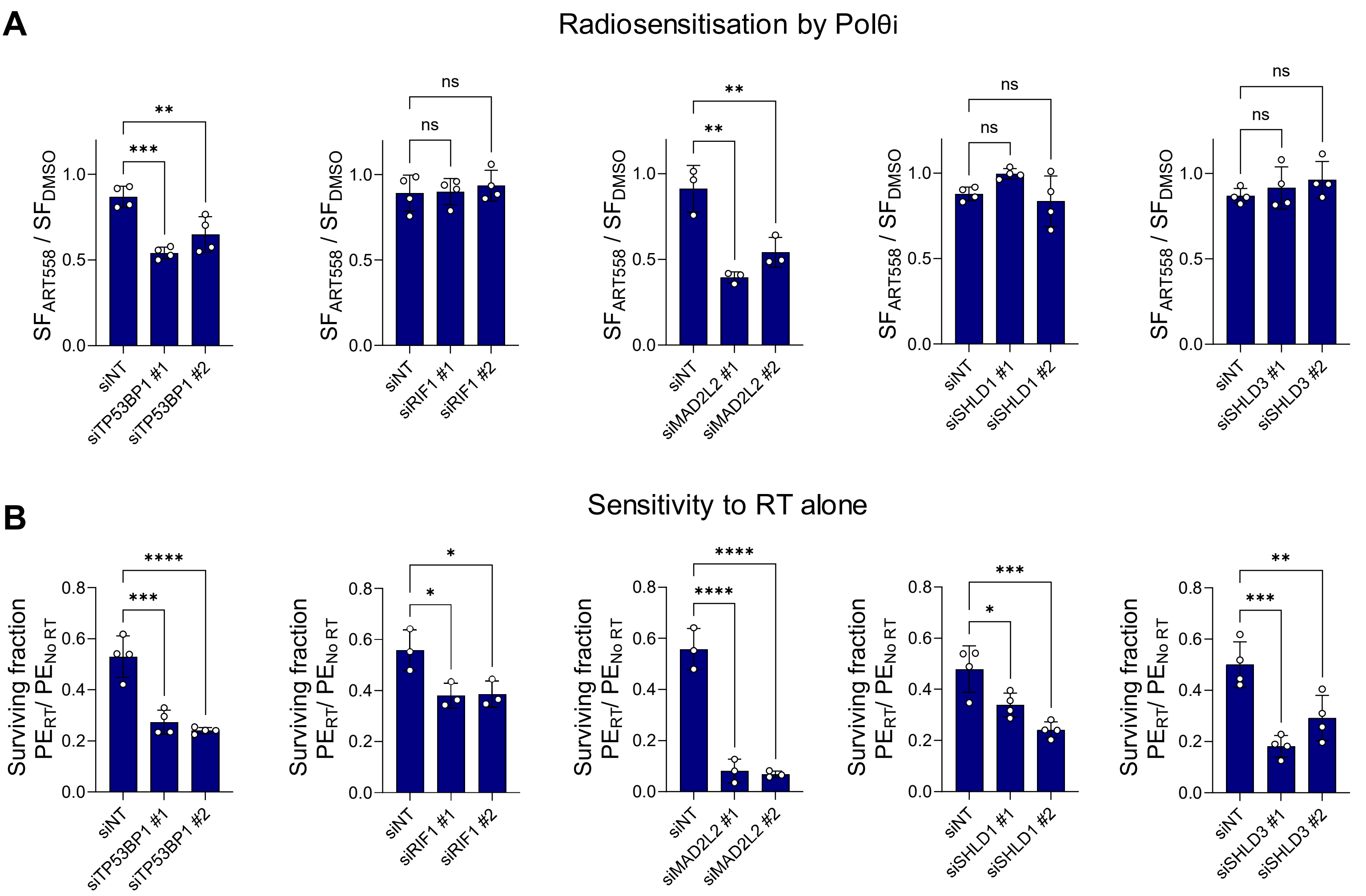

Supplementary Figure 5.

Colony-forming capacity of DU145 cells (*SHLD2*^{WT}) transfected with siRNAs against components of the TP53BP1/Shieldin axis (TP53BP1, RIF1, MAD2L2, SHLD1 and SHLD3). Cells were transfected with two independent siRNA strands per gene (#1 and #2) and subsequently treated with DMSO or 4 μmol/L Polθ inhibitor ART558 for 1 hour prior to RT (3 Gy).

(A) Radiosensitising effect of Polθ inhibitor ART558.

(B) Sensitivity to RT in the DMSO-treated cells.

Data represent mean ± SD from at least three independent repeats. ns: not significant; *p< 0.05; **p< 0.01;***p<0.005; ***p<0.001; ****p<0.0001 (one-way ANOVA).

Supplementary Figure 6 (accompanying Figure 5).

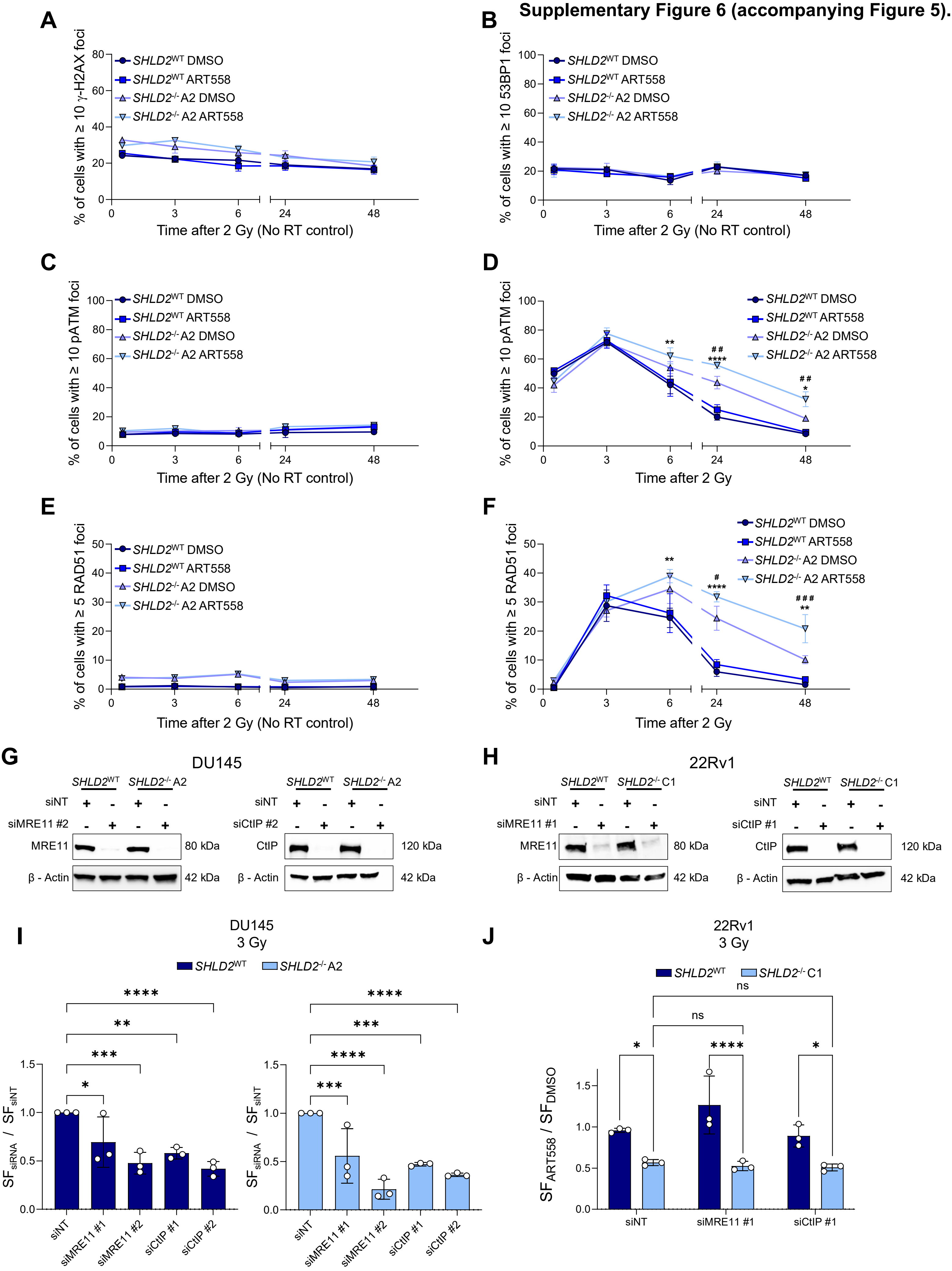

Supplementary Figure 6 (accompanying Figure 5).

(A) γH2AX and (B) 53BP1 foci dynamics, assessed by fluorescence microscopy for unirradiated controls

(C, D) pATM foci dynamics for unirradiated and irradiated conditions respectively

(E, F) RAD51 foci dynamics for unirradiated and irradiated conditions respectively

Western blots demonstrating depletion of MRE11 and CtIP in (G) DU145 cell lines and (H) 22Rv1 cell lines

(I) RT sensitivity of *SHLD2*^{WT} and *SHLD2*$^{-/-}$ A2 DU145 cells after transfection with two different siRNA strands for MRE11 and CtIP, assessed by colony formation assay. Data are expressed as the SF of siMRE11- or siCtIP-treated cells relative to the non-targeting control.

(J) Extent of radiosensitisation by Polθi upon siRNA-mediated depletion of MRE11 and CtIP in *SHLD2*^{WT} and *SHLD2*$^{-/-}$ C1 22Rv1 cells, assessed by colony formation assay.

Data are representative of three independent experiments showing mean ± SD from triplicate wells. Statistical significance was calculated with two-way ANOVA and Tukey's post-hoc test. In (D, F) asterisks and dashes refer to the comparison of DMSO-treated *SHLD2*^{WT} *vs* *SHLD2*$^{-/-}$ cells, and ART558-treated *SHLD2*^{WT} *vs* *SHLD2*$^{-/-}$ cells, respectively. # or *p<0.05; # # or **p<0.01; # # # or ***p< 0.001; # # # # or ****p< 0.0001. ns = not significant.

Supplementary Figure 7 (accompanying Figure 6).

Mouse xenograft sample	Allele 1 (bp)	Allele 2 (bp)	Predicted frameshift (%)	R^2
DU145 clone A2	-1	-4	96	0.97

Supplementary Figure 7 (accompanying Figure 6).

Confirmation of *SHLD2* KO status in the mouse xenografts. bp: base pair. (+) or (-) indicates an insertion or deletion respectively.
